# Molecular characterisation of *Pinus sylvestris* (L.) in Ireland at the western limit of the species distribution

**DOI:** 10.1186/s12862-023-02181-3

**Published:** 2024-01-23

**Authors:** Samuel Belton, Philippe Cubry, Jenni R. Roche, Colin T. Kelleher

**Affiliations:** 1 DBN Plant Molecular Laboratory, National Botanic Gardens of Ireland, Glasnevin, Dublin, Ireland; 2 DIADE, Univ de Montpellier, CIRAD, IRD, Montpellier, F-34090 France; 3 National Parks and Wildlife Service, Department of Housing, Local Government and Heritage, 90 King Street North, Smithfield, Dublin, Ireland

## Abstract

**Background:**

Scots pine (*Pinus sylvestris* L.) underwent significant population declines across much of northwest Europe during the mid-to-late Holocene and was thought to have become extirpated in Ireland from about 400 AD. However, most extant populations are plantations reintroduced from Scotland. Others are naturalised therefrom and one in Western Ireland is a putative relict. In this paper, Scots pine in Ireland are genetically described for the first time.

**Results:**

Using two mitochondrial (mtDNA) loci, eight chloroplast (cpSSR) and 18 nuclear (nSSR) loci, the genetic composition and diversity of 19 Irish Scots pine populations is described and compared to other European populations. All trees sampled in Ireland were fixed for mitotype *a*, which is the most common across northwest Europe. By contrast, cpSSR (*H*_CP_ = 0.967) and nSSR (*H*_e_ = 0.540) variation was high, and comparable with estimates for other regions across the species range. Differentiation at both sets of loci were similarly low (cpSSR *F*_ST_ = 0.019; nSSR *F*_ST_ = 0.018), but populations from continental Europe were significantly differentiated from all Irish populations based on nSSR variation.

**Conclusions:**

All Irish Scots pine are likely part of a common Irish-Scottish gene pool which diverged from continental Scots pine following post-glacial recolonisation. A high genetic diversity and an absence of evidence of inbreeding suggests the regional decline of Scots pine did not critically reduce allelic variation. The post-glacial relationship between Irish and Scottish pine is discussed, and a suggestion from recent palaeoecological work that reintroduced Scots pine be managed as a native species is now further supported by genetic data.

**Supplementary Information:**

The online version contains supplementary material available at 10.1186/s12862-023-02181-3.

## Background

Scots pine (*Pinus sylvestris* L.) occupies an enormous longitudinal range of about 14,000 km and is the only pine which is native to Ireland and Britain [1, 2]. Its distribution extends from Spain and Ireland (7°W) to the eastern coast of Siberia (138°E), and from 37°N in Spain to over 70°N in Norway [3–5]. Across this range strong local adaptation and clinal variation is reflected in efficient purifying selection in genic regions, although linkage disequilibrium (LD) decays quickly and there is high variation at neutral loci [6, 7]. A high level of shared polymorphisms at these loci and a large effective population size is a function of the highly efficient gene flow displayed by this species [6]. Scots pine is a non-specialist, pioneer species which is most competitive in poorer, marginal soils [3, 8]. Economically, it is one of the most commercially important species in Europe [9]. In Ireland, it occupies *c.* 1.2% of total forest area [10].

The post-glacial history of Scots pine is complex because of its broad range and high adaptability [11–13]. Unlike most broadleaf species, Scots pine persisted outside of the Mediterranean peninsulas during the last glacial maximum (LGM) [14–16] and evidence exists which indicate that some conifers are likely to have survived in cryptic northern refugia adjacent to glacial fronts [17, 18]. However, the extent to which these may have contributed to the current distribution of Scots pine in northwest Europe is unclear and most evidence converges on a scenario in which it was recolonised after the LGM from a southern direction, most likely from the largest refugia in the Iberian Peninsula, Italy or the Balkans, initially forming pioneer forests with *Betula* spp. [16, 19, 20].

Scots pine arrived in southern England *c*. 11,470 cal BP (calibrated radiocarbon years before present, as given in the literature) and in northern Scotland by 4,470 cal BP [1, 5, 21]. A presence in southwest Ireland is recorded at 10,740 cal BP [5, 22], though its arrival is thought to have been from across the Celtic Sea rather than through Britain [21, 23]. In Ireland, Scots pine became dominant in western and upland sites in the early Holocene. In north-central Ireland, its occurrence was more limited due to competition from *Ulmus*, *Quercus* and *Corylus*, although isolated populations on lowland peatland sites persisted [24]. By 4,500 cal BP, it was mainly restricted to mountains and raised bogs, where it became further displaced by blanket peat formation and wetter hydrological conditions, respectively [1, 24, 25]. This was part of a wider contemporaneous decline of the species across northwest Europe [26–28].

Scots pine continued to persist in isolated pockets [29, 30], but was extirpated from multiple countries in northwest Europe during the late Holocene [20, 24, 31, 32]. This was assumed to have occurred in Ireland by *c*. 1,600 cal BP, but recent evidence from a putatively native relict Scots pine population (Rockforest) in western Ireland has challenged this assumption [5, 33]. Scots pine survived in Scotland [34], and other relict populations may occur elsewhere in Britain [35, 36]. In Ireland, many non-relict populations occur which are the result of 19th and 20th century plantations from Scotland. However, some are naturalised and support vegetation communities with similarities to fossil pinewood assemblages from Ireland and extant native pinewoods elsewhere in northwest Europe [37]. Scots pine has been included in the Irish Native Woodland Scheme [38] and it has been recommended that reintroduced populations be managed as a native species [37, 39].

The relatively extensive ecological and palaeological characterisation of Irish pinewoods means that a genetic analysis is now long overdue, as concluded by other works [33, 40]. This will help to inform conservation approaches, which are important as there is growing evidence illustrating a high vulnerability of the species to climate change, and extreme shifts in its distribution range are predicted [41–45]. In this paper, Irish Scots pine are genetically characterised for the first time. In doing so, the question of whether the regional decline in Scots pine has resulted in critically low levels of genetic diversity is addressed. Results also shine light on the genetic composition and origins of Scots pine in Ireland.

## Materials and methods

### Plant material and DNA extraction

**Fig. 1:**
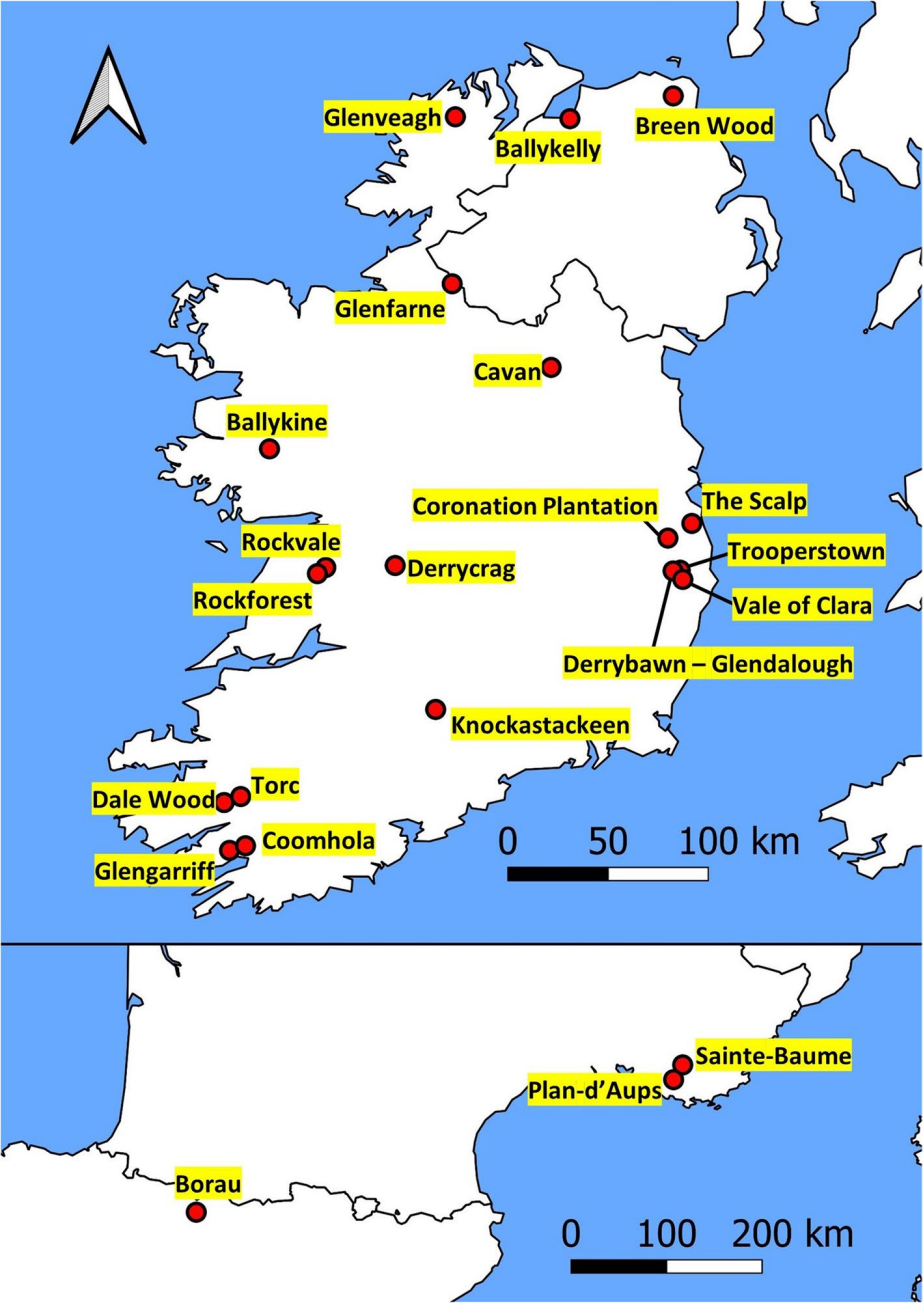
Locations/provenances of Scots pine sampling populations used in the current study

Sixteen Irish populations detailed from previous ecological surveys were selected for sampling [8, 46], along with three additional northern populations to improve geographical coverage (Fig. 1). The origin and planting date of each, where known, are given in Table S1. Material from two French and one Spanish population were also sampled, as well as four Scottish origin and three Norwegian origin genotypes. Spanish samples were collected from a native population, whereas the French samples were collected from naturalised populations from an unknown origin. The four Scottish genotypes came from a breeding programme and neither them nor the Norwegian samples were collected from discrete sampling populations. DNA was extracted as described in Belton et al. [47].

### Mitochondrial polymorphism

High resolution melting analysis (HRM) [48] was used to study mitochondrial DNA (mtDNA) variation at the *nad7* intron1 and *nad1* B/C loci [15, 49]. The former was targeted using primers H and I from Soranzo et al. [49], and the latter was targeted with internal primers (Nad7_indel_F: GCTGCCCCATTCAATTACAC; Nad7_indel_R: ATAAGGAGAGGATGCGGAAG) designed from sequences published by Pyhäjärvi et al. [15]. PCRs were performed using a QIAGEN Type-it HRM PCR kit (cat. No. 206,544) with 1 µl (5 ng) DNA working solution, 1 µl DNase-free water and 3 µl master mix on a QIAGEN Rotor-Gene Q machine using a default HRM protocol. Fluorescence was monitored continuously with an end-point melting range of 65–95 °C and alleles were called manually using the manufacturer’s software.

### Chloroplast SSR analysis

Eight mononucleotide repeat chloroplast SSRs (cpSSRs) were selected from the literature [50–52]. These were combined into two multiplex reactions (Table S2). PCRs were prepared using a Type-it Microsatellite PCR kit (QIAGEN; cat. No. 206,243), with each comprising 3 µl Type-it master mix, 2.5 µl (25 ng) DNA working solution, 1 µl primer mix and 3.5 µl DNase-free water. PCR conditions were set according to the kit quick protocol. Allele size was determined using the STRand software (UCDavis Veterinary Genetics Laboratory, Davis, USA) after electrophoresis on an ABI 3730 DNA analyser. The Scottish and Norwegian genotypes as well as one French population (Plan-d’Aups) were not included in this analysis.

### Nuclear SSR analysis

In total, 18 nuclear SSRs (nSSRs) were selected from the literature and arranged into three multiplexes [49, 51, 53–57] (Table S3). Each PCR contained 4.5 µl DNA working solution (22.5 ng), 1 µl primer mix, 7.5 µl Type-it master mix and 2 µl DNase-free water. PCRs and allele calling were the same as for the cpSSR analysis.

### Data analysis

#### Genetic diversity and differentiation (cpSSR)

Haplotypes and the frequencies of their private occurrences were determined using a custom R (v.4.2.0) script (Additional File 1), using the R packages tidyr (v.1.3.0) [58] and adegenet (v.2.1.5) [59, 60]. Adegenet was also used to handle all raw allelic data prior to statistical analyses. Genetic diversity was calculated according to Nei’s [61] unbiased genetic diversity index (*H*_CP_) using the software Haplotype Analysis (v.1.05) [62]. Allelic richness was calculated by rarefaction to seven alleles using the R package PopGenReport (v.3.0.4) [63, 64]. Genetic distance between individuals was calculated according to Nei [65] and Bruvo et al. [66] using the R package poppr (v.2.9.3) [67, 68].

Differentiation estimates (*F*_ST_) were calculated according to Nei [69] and Weir and Cockerham [70] using the R package hierfstat (v.0.5-7) [71]. Estimates of statistical significance were derived by bootstrapping using the R package mmod (v.1.3.3) [72]. Confidence interval (CI) values were, in some cases, converted to *p* values according to Altman and Bland [73]. Variation components of the data were determined using an Analysis of Molecular Variance (AMOVA), with significance determined from 1000 permutations of the data using the poppr wrapper of the AMOVA function in the R package ade4 (v.1.7–18) [74].

#### Genetic diversity and differentiation (nSSR)

Tests for LD were performed by determining the pairwise index of association (*r̄*_*d*_) [75] for each of 999 permutations of the data using poppr. Tests for departure from Hardy-Weinberg equilibrium (HWE) were performed using the exact test of HW proportions based on 1000 Monte Carlo permutations of alleles [76], as implemented in the R package pegas (v.1.0–1) [77]. As some nSSR loci were markers transferred from other species, a high frequency of null alleles was expected. Frequencies were estimated according to the expectation maximisation algorithm [78] implemented in the R package version (v.1.1.7) of GENEPOP [79]. Population genetic diversity was inferred by calculating expected heterozygosity (*H*_*e*_) using adegenet. Private alleles were identified using poppr, and allelic richness calculated by rarefaction to 14 nSSR alleles.

*F*_ST_ analysis was computed as before, whereas differentiation was also estimated based on allele size variance (*R*_ST_) according to Slatkin [80] using pegas. Inbreeding coefficients (*F*_IS_) were calculated according to Nei [69] and Weir and Cockerham [70]. The effect of null alleles on *F*_IS_ was estimated using a Bayesian approach implemented in the programme INEST (v.2.0) [81]. Specifically, a null allele model was compared against a model which also includes *F*_IS_ to describe the data. This procedure is outlined in detail in Belton et al. [82].

Genetic distance between individuals was again calculated using Nei’s and Bruvo’s genetic distance using poppr. As the latter assumes a stepwise mutation model (SMM), some loci were removed because they did not fulfil this assumption. Using the programme ML-RELATE [83], relatedness (*r*) was also estimated using a maximum likelihood (ML) procedure which accommodates null alleles [84]. In practice, *r* was estimated after readjusting allele frequencies according to the presence of null alleles, after which *r* was estimated under ML. ML-RELATE was also used to calculate the likelihood of four categories of pedigree relationship (unrelated, half-sibling, full-sibling and parent - offspring).

#### Analysis of population structure (cpSSR and nSSR)

The main genetic and spatial structure of the cpSSR haplotypes was investigated by performing a Spatial Analysis of Molecular Variance (SAMOVA) using SAMOVA (v.2.0) [85]. This approach, which attempts to maximise differentiation between groups of populations, was performed with and without geographic data. Simulations were conducted using default parameters for SSR data, with *K* ranging from two to 10 and with the annealing process for each repeated 100 times. Sub-population structure based on nSSR variation was analysed using STRUCTURE (v.2.3.4) [86], which was run across 20 CPU cores via the programme StrAuto (v.1.0) [87]. The admixture model with correlated allele frequencies was tested using sample location information (LOCPRIOR). The MCMC simulation involved a burn-in of 100,000 and 500,000 retained iterations for each of *K* = 15 inferred clusters, with 20 replications for *K*. As STRUCTURE is sensitive to deviations from HWE, simulations were repeated after adjusting for null alleles (removal of loci displaying null allele frequencies ≥ 10%). The optimal value for *K* was inferred using the Δ*K* method [88] and replications were merged using the Greedy algorithm in the programme CLUMPP (v.1.1.2) [89]. The R implementation of TESS3 (tess3r v1.1.0) was also used to infer population clusters with the use of spatial information. TESS3 is a non-model-based approach which uses matrix factorisation instead of Bayesian inference and is not sensitive to LD or deviations from HWE [90]. *K* = 1 to *K* = 20 populations were tested, each replicated 40 times. The optimal value for *K* was then determined by selecting the value with the lowest cross-validation score, as recommended by the programme authors. Finally, in addition to pairwise *F*_ST_ and *R*_ST_ comparisons, population sub-division was further investigated by performing a Principal Coordinates Analysis (PCoA) based on Nei’s genetic distance using adegenet.

## Results

### Mitochondrial variation

All samples exhibited the same *nad7* intron 1 allele. Only Spanish trees contained a different allele from other provenances at the *nad1* B/C intron. Samples from Ireland, Scotland, France and Norway were therefore all fixed for the most common European mitotype, which is mitotype *a*, whereas the Spanish samples analysed were all fixed for mitotype *c* (data not shown).

### Chloroplast variation

**Fig. 2:**
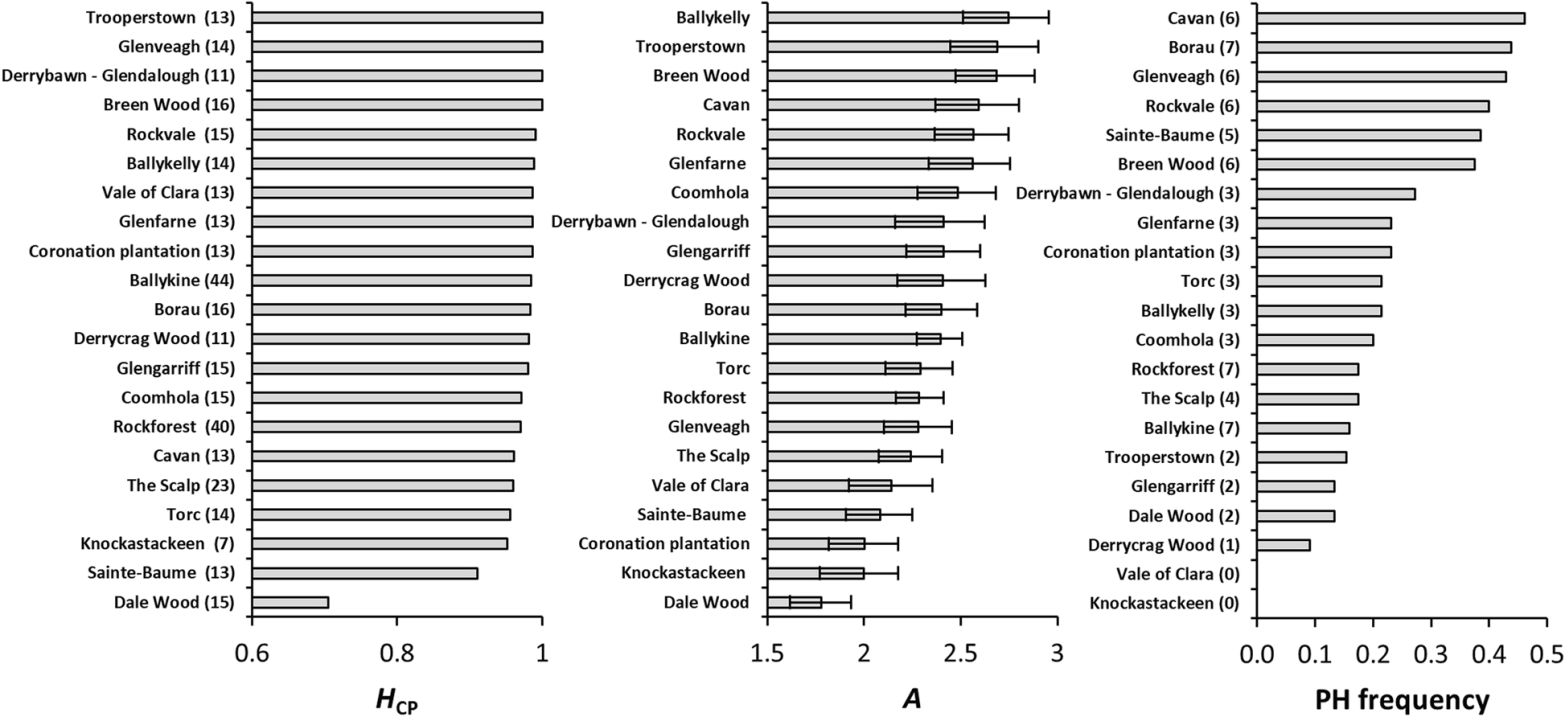
Chloroplast genetic diversity (*H*_CP_), allelic richness (*A*) and the frequency of private haplotypes (PH) for each Scots pine population (excluding Norwegian and Scottish samples). *A* was calculated based on rarefaction down to seven cpSSR alleles. Values in brackets in the *H*_CP_ and PH frequency plots are sample number and the number of detected PHs, respectively. Error bars on *A* values are 95% CIs derived from 1000 bootstrap permutations of the data

Between two and seven alleles were identified per cpSSR locus (Table 1). The distributions of allele size variants were unimodal for each, with minor alleles belonging to the extremes of the allele size ranges. Variation at the cpSSR level was very high; a total of 134 haplotypes were detected, 122 of which occurred in Ireland (Table S1). The highest frequency (6.61%) for any one haplotype was H15. An average of 3.76 private haplotypes were detected, with frequencies being highest for Cavan and lowest for Vale of Clara and Knockastackeen, which harboured none (Fig. 2). Allelic richness was highest in Ballykelly and lowest in Dale Wood, which also had the lowest *H*_CP_ (Fig. 2). Overall *H*_CP_ was 0.965 (± 0.063 S.D.), or 0.967 (± 0.065 S.D.) when removing continental populations. Altogether, the number of different alleles and genetic diversity was lowest in Dale Wood, whereas no single population stood out for having a particularly high level of either.

**Table 1:** Diversity and differentiation statistics of the eight cpSSR markers used to genotype Scots pine (*Pinus sylvestris*) individuals (*n* = 348). Statistics include number of alleles (N), allelic richness (*A*), intra-population diversity (*h*_S_), total diversity (*h*_T_), diversity which apportions between populations (*F*_ST_), with (Nei) and without adjusting for sample size (WC). *F*_ST_ values which are marked with an asterisk are significantly different from zero (*p* ≤ 0.05)

	*N*	Allele size range	*A*	*h*_S_	*h*_T_	*F*_ST_(Nei)	*F*_ST_(WC)
Pt1254	5	59–63	1.400	0.384	0.388	0.011	0.012
Pt41093	2	73–74	1.118	0.124	0.125	0.013	0.008
Pt26081	3	107–109	1.385	0.360	0.363	0.008	0.016
Pt15169	7	123–129	1.709	0.707	0.726	0.027	0.026
Pt30204	6	137–142	1.670	0.671	0.712	0.058*	0.051**
Pt71936	4	142–145	1.281	0.275	0.281	0.021	0.016
Pt36480	6	142–147	1.618	0.624	0.640	0.026	0.027
Pt87268	5	161–165	1.257	0.273	0.273	0.000	0.000
mean	4.75	-	1.430	0.427	0.439	0.020*	0.019*

Mean Nei genetic distance between individuals calculated at the population level ranged from 0.359 (Dale Wood) to 0.811 (Rockvale), or from 0.176 (Dale Wood) to 0.300 (Ballykelly) when using Bruvo’s genetic distance (Figure S1). As the latter assumes a SMM, it may be a more realistic description of haplotype relatedness compared to Nei’s standard genetic distance, which assumes only an Infinite Alleles Model. Overall *F*_ST_ was very small but significantly greater than zero (*F*_ST_ = 0.020, 95% CI: 0.001–0.041, or *p* = 0.05; Table 1). This significance was mainly attributable to Pt30204, which was the only cpSSR locus which displayed an *F*_ST_ value which was significantly greater than zero (Table 1). Only a sample size-independent estimate (*F*_ST_ = 0.019, 95% CI: 0.001–0.037, or *p* = 0.046) was significant when just Irish populations were considered. Pairwise *F*_ST_ comparisons showed that this was almost entirely due to differentiation between Dale Wood and all other populations (Figure S2). AMOVA showed that only 2.47% of total variation was between populations (or 2.41% when only Irish populations were considered; Table 2).

**Table 2:** Results of an AMOVA based on cpSSR variation. AMOVA results are given based on all sampled trees (All) and based on trees sampled in Ireland only

**Components (All)**	**d.f**	**SSD**	**Variance**	**Variance (%)**	***p*** **value **
Between populations	20	97.146	0.087	2.472	0.001
Within populations	327	1123.182	3.435	97.528	< 0.001
Total	347	1220.328	3.522	100.000	
**Components (Ireland)**	**d.f**	**SSD**	**Variance**	**Variance (%)**	***p*** **value**
Between populations	18	87.461	0.085	2.406	0.002
Within populations	300	1035.893	3.453	97.594	< 0.001
Total	318	1123.354	3.538	100.000	

### Nuclear variation

Six nSSR loci had null allele frequencies exceeding 10% (Table 3) and subsequent analyses were performed before and after removing them. All loci were considered unlinked, as no significant LD (*p* ≤ 0.05) was found for 3,672 pairwise comparisons after correcting for familywise error (Holm and Bonferroni). Following Bonferroni correction, significant departure from HWE was detected for 20 out of 432 cases, corresponding to HWE departures in nine populations (Table 4). This dropped to two out of 288 tests after adjusting for null alleles. Null alleles also inflated initial *F*_IS_ estimates (Table 4), and only Coomhola displayed a significant (albeit low) null allele-adjusted *F*_IS_ value. Overall, *F*_IS_ for Ireland was very low, at 0.022 (95% highest density interval: 0.0049–0.0397).

**Table 3:** Summary statistics for the 18 nSSR loci used to genotype Scots pine. Abbreviated values from left to right include the total detected alleles (N), allelic richness (*A*), observed heterozygosity (*H*_*o*_), expected heterozygosity (*H*_*e*_), inbreeding coefficient (*F*_IS_) and genetic diversity which apportions between populations (*F*_ST_). *F*_IS_ and *F*_ST_ values are calculated according to Nei (1987) and Weir & Cockerham (1984). The latter are unbiased by sample size differences. *F*_IS_ and *F*_ST_ values in bold are significantly greater than zero. (*p ≤ 0.05; **p ≤ 0.01; p*** ≤ 0.001). Loci with null frequency values in bold were removed from analyses where indicated

Locus	*N*	Allele size range	*A*	*H*_*o*_	*H*_*e*_	*F*_IS_(Nei)	*F*_IS_(WC)	*F*_ST_(Nei)	*F*_ST_(WC)	Null frequency
PtTX3116	26	116–278	1.803	0.458	0.869	0.453***	0.459***	0.038**	0.030***	**0.175**
PtTX3032	49	275–512	1.916	0.874	0.954	0.088***	0.076***	0.012	0.010*	0.026
psy119	5	310–322	1.130	0.100	0.104	0.013	0.019	0.035	0.027	0.005
SPAC11.6	45	108–220	1.881	0.615	0.926	0.346***	0.328***	0.016	0.016**	**0.135**
psy117	8	215–229	1.642	0.230	0.691	0.672***	0.659***	0.030*	0.030**	**0.251**
SPAC12.5	30	122–190	1.879	0.901	0.914	-0.016	-0.010	0.020*	0.021***	0.019
SPAG7.14	55	174–244	1.900	0.808	0.944	0.117***	0.126***	0.025***	0.024***	0.037
PtTX3107	12	144–171	1.723	0.379	0.765	0.465***	0.503***	0.009	0.004	**0.182**
PtTX4001	15	196–226	1.755	0.762	0.781	0.014	0.005	0.027*	0.023**	0.021
PtTX4011	9	231–277	1.530	0.441	0.583	0.227***	0.207***	0.062***	0.050***	0.075
psy118	7	287–317	1.134	0.111	0.120	0.058	0.068	0.009	0.009	0.010
psy12	4	197–206	1.303	0.255	0.302	0.140*	0.141*	0.024	0.019	**0.769**
psy125	2	212–215	1.042	0.038	0.043	0.087	0.086	0.019	0.034	**0.312**
psy136	5	244–256	1.098	0.122	0.117	-0.038	-0.045	0.000	0.000	0.000
psy142	6	165–175	1.648	0.659	0.677	-0.002	0.011	0.020	0.018	0.030
psy144	5	164–175	1.148	0.120	0.144	0.043	0.078	0.103*	0.105**	0.014
psy157	6	184–199	1.388	0.369	0.414	0.036	0.050	0.086***	0.066***	0.021
SPAC11.4	20	132–170	1.811	0.842	0.852	-0.015	-0.015	0.035***	0.025***	0.016
Mean	17	-	1.541	0.449	0.567	0.149***	0.156***	0.032***	0.028***	0.117

**Table 4:** Geographic coordinates for Scots pine sampling populations. Also indicated is the number of trees sampled per population (N), the frequency of private alleles (Pa) detected, Nei’s inbreeding coefficient (*F*_IS_) and null allele-adjusted *F*_IS_ (*F*_IS_INEST). Values which are significantly different from zero are marked with asterisks (*p ≤ 0.05; **p ≤ 0.01; p*** ≤ 0.001). *F*_IS_ values in bold indicate significant departure from HWE and *F*_IS_INEST values in bold indicate that an inbreeding model was favoured over a null allele model

Population	Longitude	Latitude	*N*	Pa	*F*_IS_	*F*_IS_INEST
Ballykelly	-7.0487	55.0434	13	0.0000	0.2121**	**0.0824**
Ballykine	-9.3461	53.5585	43	0.0078	0.2025***	**0.0465**
Borau	-0.5882	42.6581	12	0.0278	0.1831**	**0.0387**
Breen Wood	-6.2376	55.1364	13	0.0256	0.2024**	**0.0418**
Cavan	-7.2172	53.9289	13	0.0085	0.1733*	**0.0273**
Coomhola	-9.4662	51.7759	13	0.0085	0.1959**	0.0781*
Coronation plantation	-6.3598	53.1544	14	0.0040	0.1905**	**0.0589**
Dale Wood	-9.6277	51.9678	13	0.0043	0.2241*	**0.0293**
Derrybawn - Glendalough	-6.3268	53.0065	16	0.0208	0.2395***	0.0707
Derrycrag Wood	-8.3941	53.0420	9	0.0000	0.0979	0.0399
Glenfarne	-7.9691	54.3065	14	0.0119	0.2099*	0.0203
Glengarriff	-9.5807	51.7538	15	0.0000	0.1956*	0.0482
Glenveagh	-7.9437	55.0557	14	0.0000	0.1332*	**0.0430**
Knockastackeen	-8.0917	52.3968	7	0.0000	0.1454*	0.0403
Norway^a^	8.5	60.4	3	0.0370	0.2381*	0.0345
Plan-d’Aups	5.7170	43.3300	7	0.0238	0.2067*	**0.0700**
Rockforest	-8.9726	53.0022	35	0.0063	0.1622*	0.0191
Rockvale	-8.9106	53.0303	15	0.0185	0.1851*	0.0122
Sainte-Baume	5.8640	43.4525	8	0.0347	0.2551**	0.0862
Scottish (Coillte)^a^	-4.2	56.4	4	0.0000	0.1741*	0.0429
The Scalp	-6.1786	53.2176	23	0.0072	0.1784**	0.0409
Torc	-9.5079	51.9964	14	0.0040	0.2519***	0.0719
Trooperstown	-6.2736	53.0090	12	0.0093	0.1023*	**0.0489**
Vale of Clara	-6.2562	52.9674	14	0.0040	0.2201***	**0.1089**

^a^These are groups of individuals which originate from the same country but not necessarily the same forest site. Geographic coordinates for these are country level only and are given for GIS plotting

**Fig. 3:**
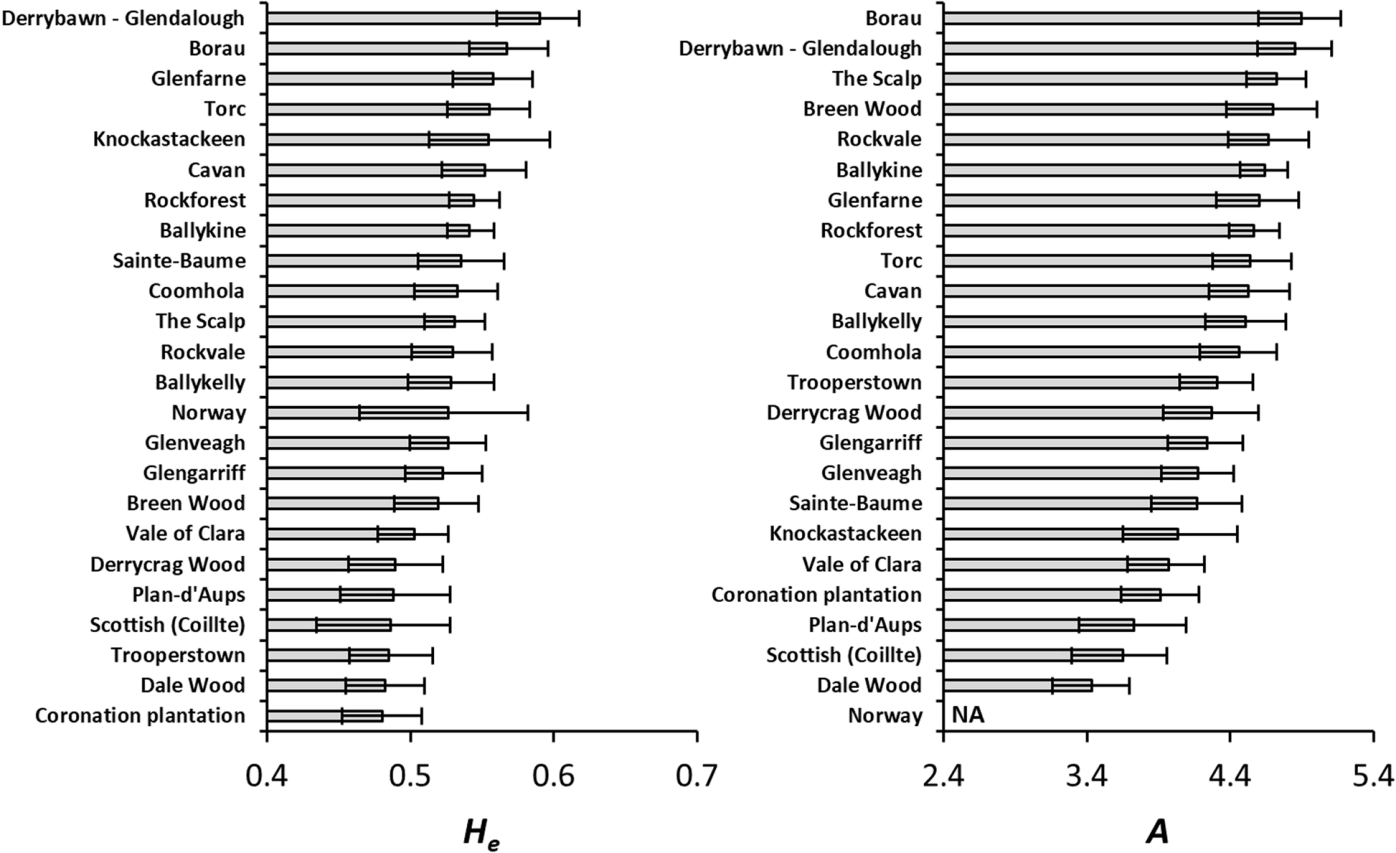
Genetic diversity (*H*_*e*_) and allelic richness (*A*) based on nSSR variation in Scots pine sampling populations. *A* was calculated based on rarefaction down to 14 alleles. Error bars are 95% CIs derived from 1000 bootstrap permutations of the data

Diversity (*H*_*e*_) and allelic richness (*A*) were highest for Derrybawn – Glendalough and Borau, and lowest for Coronation Plantation and Dale Wood respectively (Fig. 3). These differences remained after adjusting for null alleles (Figure S3). For all Irish populations, *H*_*e*_ was 0.559 (or 0.540 after adjusting for null alleles).

**Fig. 4:**
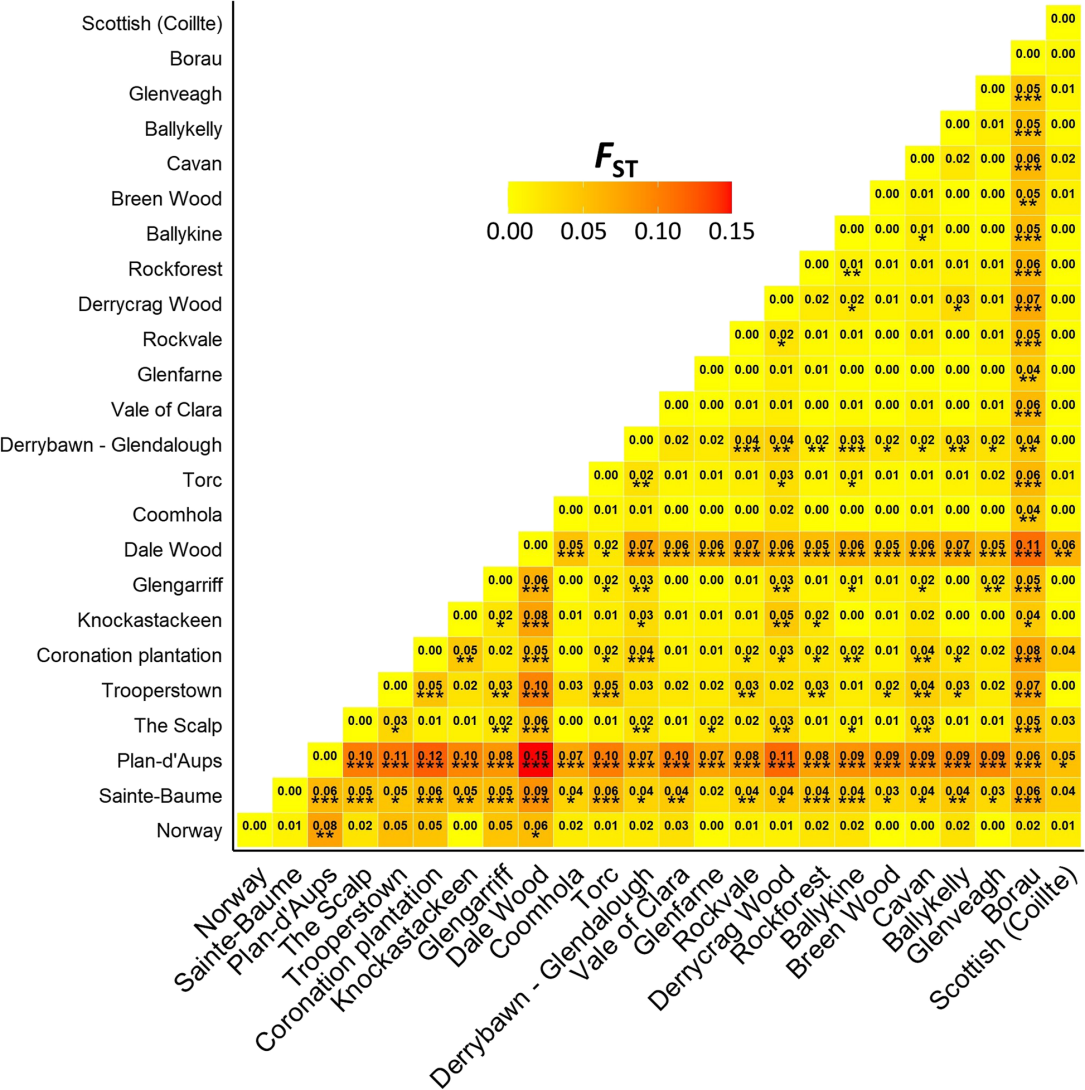
Pairwise *F*_ST_ comparisons between sampled populations. Estimates are based on variation of nSSR allelic frequency according to Nei (1987). Values which are significantly different from zero are marked with asterisks (*p ≤ 0.05; **p ≤ 0.01; p*** ≤ 0.001; *n* = 344)

Differentiation at the nSSR level (*F*_ST_ = 0.032, 95% CI: 0.022–0.037, or *p* < 0.001; Table 3) was not significantly greater than that observed for the cpSSR loci. When removing non-Irish populations, *F*_ST_ remained low but significant (*F*_ST_ = 0.018, 95% CI: 0.014–0.023, or *p* < 0.001). Null alleles did not affect these estimates. Pairwise comparisons revealed that differentiation was highest between Plan-d’Aups and Dale Wood (*F*_ST_ = 0.149, 95% CI: 0.120–0.181, or *p* < 0.001; Fig. 4). Although private allele frequency was generally highest for continental populations (Table 4), differentiation based on allele size (rather than allele frequency) was overall not significant (*R*_ST_ = 0.011, 95% CI: 0.000-0.065, or *p* = 0.518; Figure S4). AMOVA also showed that a similarly low proportion (3.84%) of variation was due to between-population differences, although the contribution of non-Irish populations appeared to be greater as removing them reduced the between-population variance to 2.73% (Table 5).

**Table 5:** Results of a AMOVA based on nSSR variation. AMOVA results are given based on all sampled trees (All) and based on trees sampled in Ireland only

**Components (All)**	**d.f**	**SSD**	**Variance**	**Variance (%)**	***p*** **value **
Between populations	23	192.973	0.214	3.837	0.001
Within populations	319	1714.158	5.374	96.163	< 0.001
Total	342	1907.131	5.588	100.000	
**Components (Ireland)**	**d.f**	**SSD**	**Variance**	**Variance (%)**	***p*** **value**
Between populations	18	139.169	0.150	2.733	0.001
Within populations	290	1546.136	5.332	97.267	< 0.001
Total	308	1685.305	5.481	100.000	

**Fig. 5:**
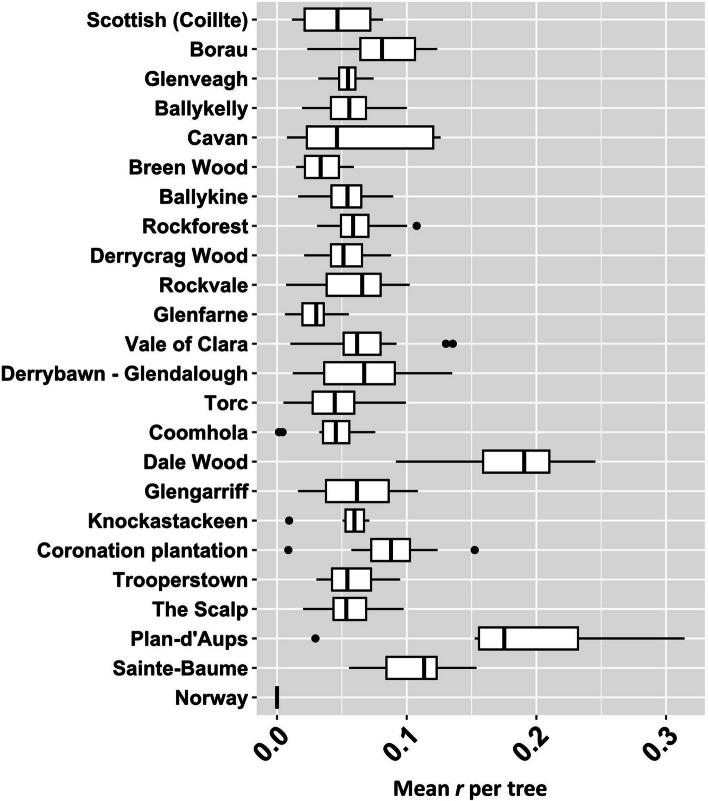
Boxplot showing mean values of *r* estimated under ML based on nSSR variation

Mean Nei genetic distance between individuals ranged from 0.463 (Coronation Plantation) to 0.670 (Torc; Figure S5a). As for cpSSR genetic distance, mean nSSR genetic distance based on allele size differences (*i*.*e*., assumption of a SMM) yielded different estimates; after removing SPAG7_14 and psy144, which violated the SMM, mean Bruvo’s genetic distance based on nSSR alleles ranged from 0.338 (Plan-d’Aups) to 0.421 (Scottish (Coillte); Figure S5b). Out of 58,996 pairwise comparisons, the distribution of ML estimates for *r* reduced rapidly, with 83.26% being 0.1 or less (Figure S6). Distributions were less skewed for Dale Wood, Plan-d’Aups and Sainte-Baume (Figure S7), populations which also showed greater mean values of *r* per tree (Fig. 5). Dale Wood and Plan-d’Aups also had the highest frequency of full and half siblings, and the lowest frequency of unrelated individuals (Figure S8).

### Population structure

SAMOVA based on cpSSR variation revealed that the main structure was between Dale Wood and all other populations (*i*.*e*., *K* = 2; *F*_CT_ = 0.0828, *p* = 0.0489), among which differentiation was not significant (*F*_SC_ = 0.0141, *p* = 0.0860; Table S4a). Glenfarne separated as a third group at *K* = 3 and Knockastackeen as a fourth at *K* = 4. At the latter, Torc also clustered with Dale Wood, whereas *F*_SC_ was negative at *K* ≥ 5. When the data were geographically constrained, results remained the same until *K* ≥ 9, after which group assignment differed (Table S4b). PCoA also showed that Dale Wood clearly separated along the main axis, which explained 59% of the variation (Figure S9), which is in agreement with pairwise *F*_ST_ and SAMOVA results. No approach showed any clear distinction between Irish and continental populations.

**Fig. 6:**
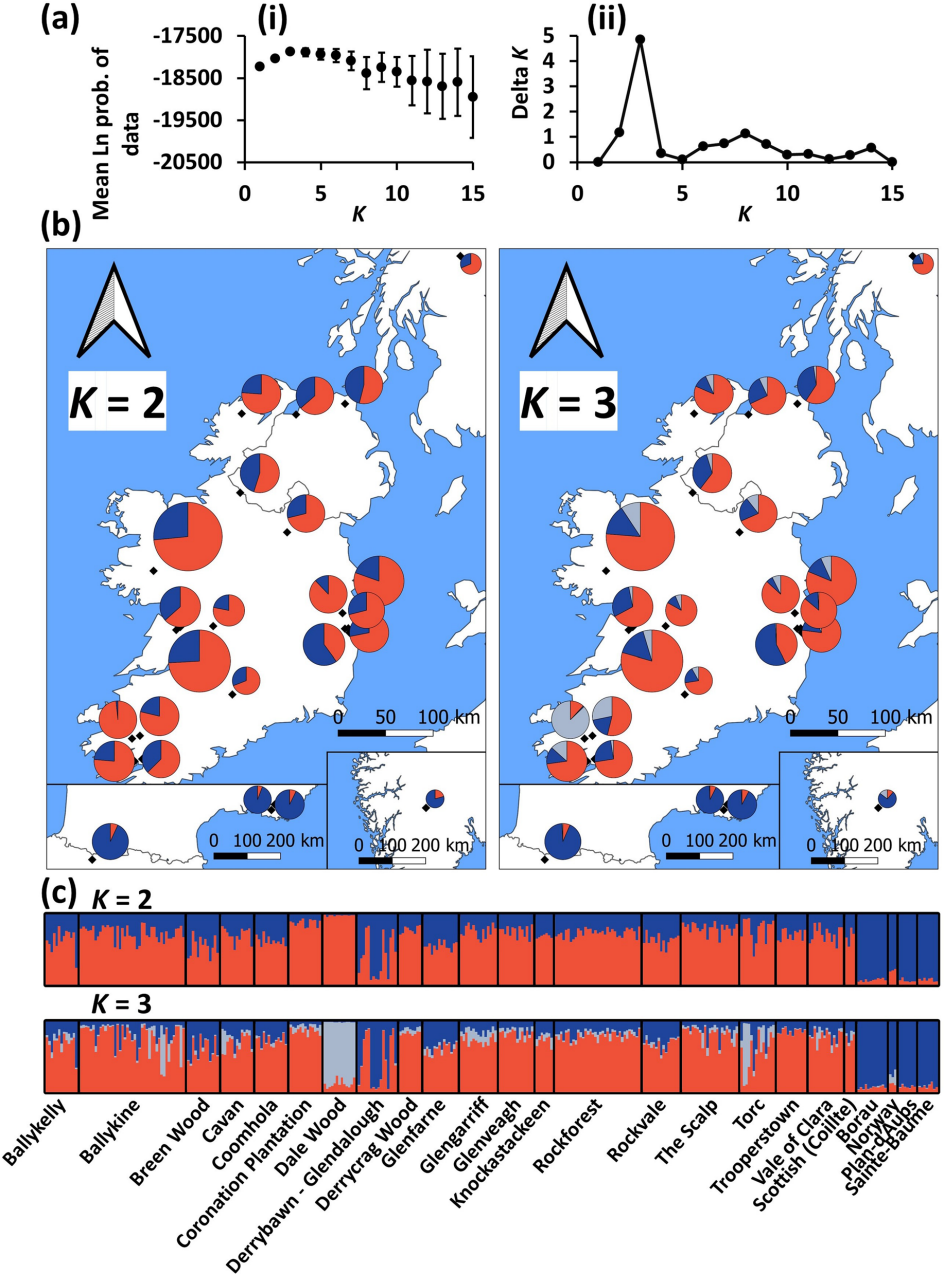
Results of STRUCTURE analysis of Scots pine nSSR data. **a **(i) Mean posterior probability values of *K* (L(*K*)), error bars are ± S.D. (ii) Delta *K* values used to infer the main structure of the data. **b** Frequency of *K* groups within each sampling population, including those located on the continent (inset). Pie charts in Scotland and Norway do not indicate an exact sampling location, but are generic origins plotted for comparison’s sake. **c** Bar plots of admixture coefficients for each sampled individual

**Fig. 7:**
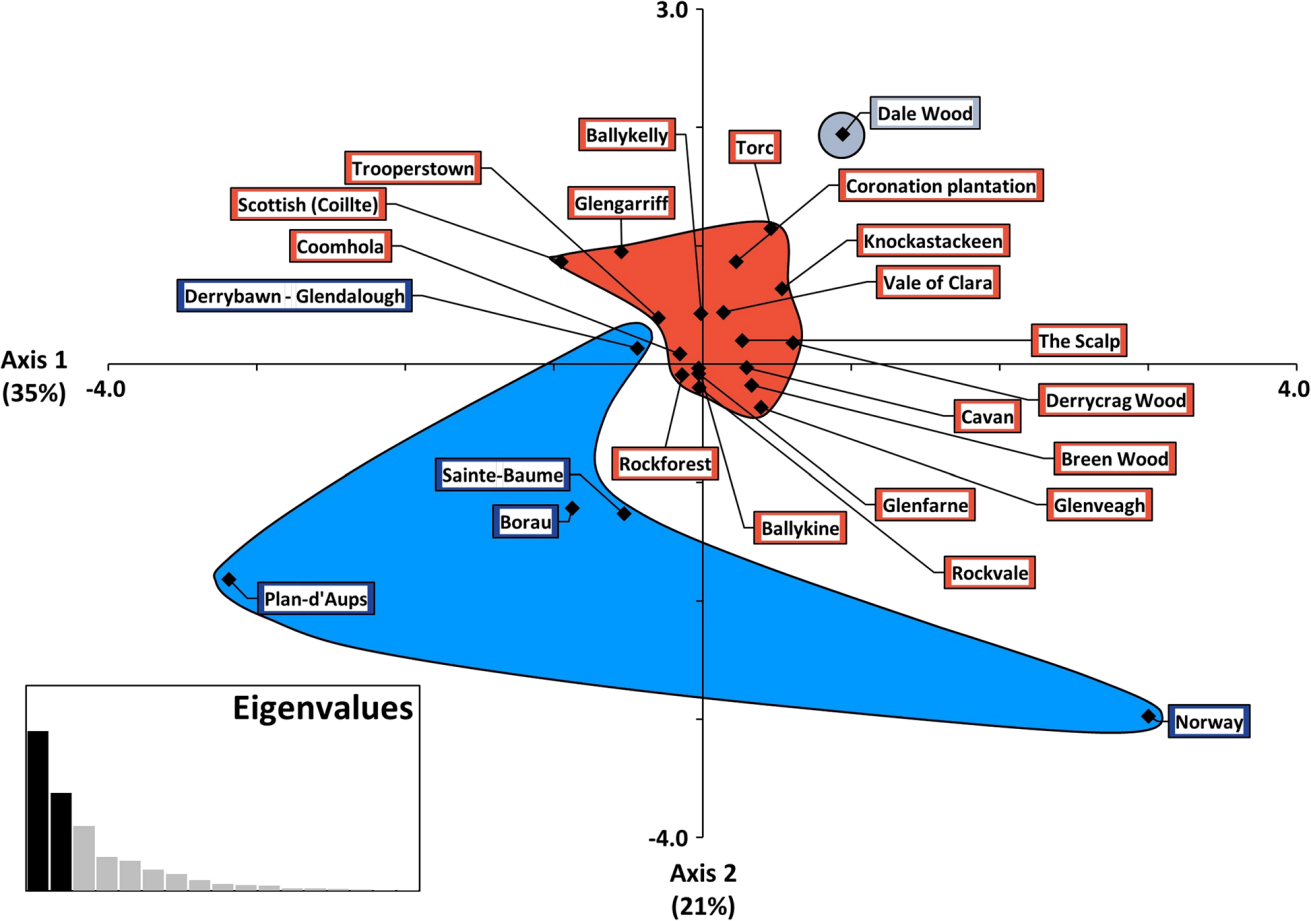
Principal Coordinates Analysis (PCoA) of the nSSR allelic composition of each Scots pine population. Shown are the first and second principal coordinates. Labels are coloured according to the STRUCTURE group (*K* = 3) that is most frequent in each population. Coloured shapes are also drawn around these points to assist in interpretation

**Fig. 8:**
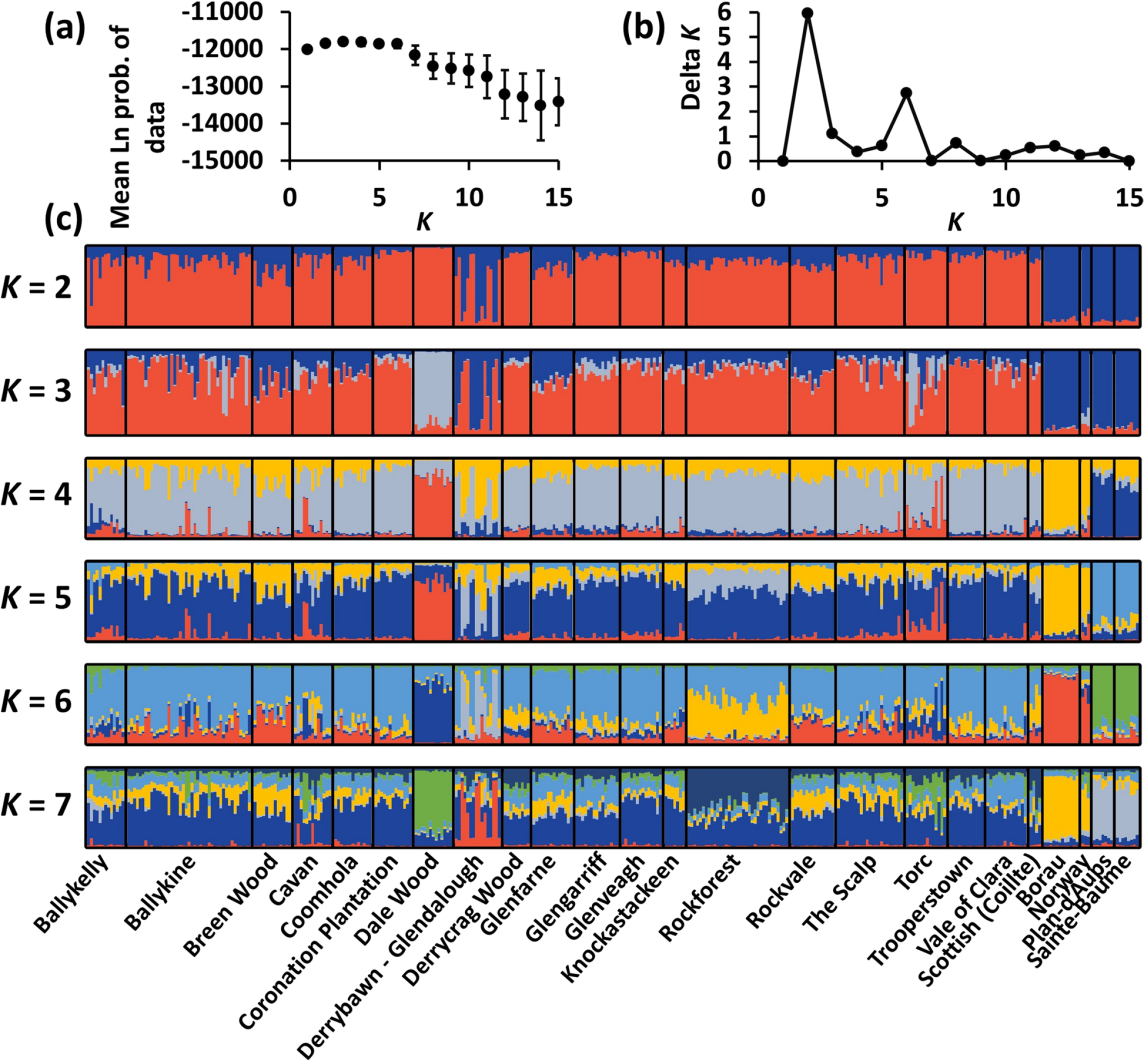
Results of a STRUCTURE analysis after removing loci (PtTX3116, SPAC11_6, psy117, PtTX3107, psy12 and psy125) with excessive null allele frequencies. **a** Mean posterior probability values for *K* (L(*K*)), error bars are ± S.D. **b** Delta *K* values. **c** Bar plots of admixture coefficients at different values of *K* for each individual

By contrast, STRUCTURE analysis of the nSSR data showed that the Irish samples and the four Scottish genotypes were clearly differentiated from the continental populations (including the three Norwegian genotypes) at *K* = 2 and *K* = 3 (Fig. 6). Derrybawn – Glendalough displayed a relatively high level of admixture with a gene pool that was most frequent on the continent, whereas Torc and especially Dale Wood in the southwest of Ireland displayed a disproportionate frequency of a third ancestral group at *K* = 3. Both axes of a PCoA plot separated the Irish and continental populations, suggesting that the main structure is described at *K* = 2 (Fig. 7). As STRUCTURE is sensitive to deviations from HWE, the analysis was repeated after adjusting for null alleles, with the optimal value for *K* indeed being inferred at *K* = 2 ancestral groups (Fig. 8, Figure S10). This was also more apparent based on a null allele-adjusted PCoA (Figure S11). At this level of structure, admixture with continental trees was inferred to be lower. Continental and several Derrybawn – Glendalough trees also separated as before, with Torc and Dale Wood becoming differentiated again at *K* = 3. Interestingly, Δ*K* was second highest at *K* = 6 (Fig. 8b), for which one genotypic group was more frequent in Rockforest trees compared to all other Irish trees (Fig. 8c). This was the case for each of *K* = 5 to *K* = 15 genotypic groups (Figure S12), indicating a very small shift in allele frequency in Rockforest trees.

TESS3, which is not sensitive to null alleles but uses spatial coordinates to assist clustering, also suggested *K* = 2 as the main structure (Figure S13). Unlike with STRUCTURE however, this separated all Irish trees such that neither Derrybawn – Glendalough, Torc nor Dale Wood were differentiated at either *K* = 2 or *K* = 3. TESS3 also did not infer any additional substructure at *K* ≥ 4 (data not shown).

## Discussion

### Genetic diversity of Irish Scots pine in a European context

The absence of any diversity at the selected mtDNA loci is in line with other works, which show that these alleles are fixed across most of the Scots pine distribution range [14, 91]. Unlike mtDNA, conifer chloroplast DNA (cpDNA) has an intrinsically higher substitution rate and is dispersed via pollen, which gives rise to much greater variation [92–95]. The variation observed here at cpSSR loci was high (Table 1). Many of these loci have been used in other works to produce diversity estimates for populations of Scots pine across its distribution range (Table S5, Table S6). *H*_CP_ for Irish Scots pine (*H*_CP_ = 0.967 ± 0.065 S.D.) was similar to a range-wide average (*H*_CP_ = 0.888 ± 0.109 S.D.; Table S5). This is probably a relatively reliable comparison, as re-estimations of *H*_CP_ for Irish trees using different subsets of cpSSR loci in accordance with different works are comparable with estimates for autochthonous pine in most regions across the distribution range (Table S6). Compared to some regions – Lithuania, Crimea, Caucasus and Asia Minor – *H*_CP_ for Ireland is even higher (Table S6).

The choice of nSSR loci has been much less standardised across works. Here, *H*_*e*_ estimates for each of 18 nSSR loci varied between 0.043 and 0.954 (Table 3), and produced an overall null allele-adjusted value of 0.540 (95% CI: 0.532–0.548) for Irish Scots pine. By contrast, Scalfi et al. [51] used just three highly variable loci (SPAC12.5, SPAG7.14 and SPAC11.4; *H*_*e*_ = 0.63–0.93) to estimate a value of 0.81 for Scots pine populations in northern Italy. *H*_*e*_ for Irish populations using this subset was 0.894 (95% CI: 0.886–0.901), and in this work, Spanish and French Scots pine did not differ from Irish trees when using the full loci set (Figure S14).

In general therefore, it appears that even though most Irish Scots pine come from reintroduced plantations, levels of genetic diversity are at least comparable – if not greater – than native pinewoods elsewhere. This may be partly due to plantations being sourced from Scotland, where diversity is particularly high (*H*_CP_ = 0.982-1) [96, 97], potentially as a result of admixture between two refugial gene pools [98].

### What is the genetic composition of Irish Scots pine?

A site in western Ireland (Rockforest, County Clare) was recently identified as a microrefugium of Scots pine, thus re-defining the natural range for the species [5, 33]. Palynological evidence indicates a continuous Scots pine presence at this site since at least 1,600 cal BP [5, 33], with macrofossil evidence pointing to a presence by at least 5,800 cal BP [39]. Pollen evidence from two locations within 40 km of Rockforest also suggests that parts of the west were colonised by *c*. 10,500 cal BP [22, 99]. As such, it appears that the Rockforest stand is a relict population of Scots pine, which previously occupied suitable sites across Ireland [21, 46]. It is therefore a good population to use for comparison with other populations of reintroduced Scots pine.

#### Insights from mtDNA loci

Scots pine’s mitochondrial genome, which tracks seed dispersal, can be divided into five mitotypes (*a*-*e*) across its entire range and can be used to infer post-LGM and phylogeographic patterns [14, 91]. Excluding the limited number of Spanish trees which were fixed for mitotype *c*, all trees in the current study were fixed for mitotype *a*. This mitotype is also found throughout Spain, and is the most common mitotype across the species range, dominating northwest and central Europe, the Balkans, western Scandinavia and most of Siberia [14, 100, 101]. Mitotype *c* is generally restricted to northeast Spain and did not expand into northern Europe following the LGM [14, 41].

Improved phylogeographic resolution is now being provided by the discovery of additional mtDNA loci. For example, Wachowiak et al. [102] showed that Scots pine in its northern range contains at least eight mitotypes. Others have elucidated the composition of the Caucasus, and revealed that mitotype *a* in the Ural region can be divided into two subtypes [101, 103, 104]. Sinclair et al. [105] used the *coxI* gene as a probe to identify RFLPs in mtDNA from native Scottish pine. They identified three mitotypes, and a western-specific mitotype was hypothesised to be evidence of post-glacial migration from Ireland. However, the sequence variation defining these RFLPs currently remains unknown.

#### Insights from cpSSR loci

Although variation at cpSSR loci was relatively high, hardly any of this was between populations (Table 2, Table S4). This is also the case between Scottish and continental populations, and it is only between European and Anatolian populations that cpSSR structure is apparent [96]. A recent establishment of northwest populations together with homogenising pollen-mediated gene flow likely explain the absence of sub-population differentiation in Europe [6, 93, 106, 107]. In this study, only Dale Wood was differentiated (Table S4, Figure S2), and this was due to very low variation (Fig. 2). This population (planted in 1829) was the most fixed for any one haplotype, presumably because it was derived from a limited seed source. Interestingly, the nearby Torc population (planted in 1880) was grouped with Dale Wood based on allelic composition at *K* = 4 cpSSR groups (Table S4).

#### Insights from nSSR loci

The between-population variation component at nSSR loci was slightly greater than at cpSSR loci (Table 5). This was due to differentiation between Irish and continental populations, with each belonging to a distinct gene pool (Fig. 6, Figure S10, Figure S13). Populations in Scotland are also differentiated from European populations at nDNA loci [98, 102], indicating that the Celtic Sea and English Channel act as relatively strong barriers to gene flow. Shifts in allele frequency giving rise to differentiation at cpSSR loci may occur more slowly due to lower variation and an absence of recombination.

Although 30 private alleles were detected in continental populations, the island and continental gene pools were characterised by variation in allele frequency (*F*_ST_; Fig. 4) rather than allele size (*R*_ST_; Figure S4). This may be partly shaped by the marginal differences in within-population relatedness observed between Irish and continental populations (Fig. 5). However, significant deviations from HWE were not detected in the continental populations whereas inbreeding was low in all populations (Table 4). Moreover, the two most differentiated populations (Dale Wood and Plan-d’Aups; Fig. 4) also harboured the highest frequency of related trees (Fig. 5). Therefore, these gene pools cannot only be a function of differences in within-population relatedness but are at least also reflective of a different demographic history.

Interestingly, geographic variability in allele frequency distributions among Scottish Scots pine has been suggested to signal historic admixture between two refugial gene pools during post-glacial recolonisation [98]. That admixture between the observed *K* = 2 gene pools here was greater in Irish trees is consistent with this, as most Irish Scots pine are recent reintroductions from Scotland. Under this scenario, the lower within-population relatedness observed in Irish trees can also be expected. Łabiszak and Wachowiak [108] recently estimated admixture based on variation at 9,760 nuclear SNPs in 62 Eurasian Scots pine populations. Two of these were located in south-central France and both comprised of only one ancestral group when both *K* = 3 and *K* = 5 were inferred. This was the lowest admixture of all studied populations. The higher mean-relatedness in the two French populations sampled here may therefore be explained by Scots pine in this region originating from only one refugial gene pool (Figs. 5 and 6, Figure S10).

Although the Irish populations seem to be part of the same meta-population, some trees located in Derrybawn – Glendalough appeared to be of continental origin (Fig. 6, Figure S10). A mixed origin of this population may account for its relatively high level of genetic variation (Fig. 3). Additional nuclear substructure at *K* = 3 was also observed in two south-western populations (Fig. 6, Figure S10). This was defined by the increased occurrence of a third ancestral group at a > 50% frequency in all Dale Wood trees, which may reflect the relatively fixed allele frequency in this population, as indicated by its high *F*_ST_ and low diversity estimates (Figs. 2, 3 and 4, Figure S2). To a lesser extent, several trees in the nearby Torc population also possessed this third group (Fig. 6, Figure S10).

At *K* = 2 and *K* = 3, Rockforest trees shared genotypic proportions which were indistinguishable from almost all other Irish populations (Fig. 6, Figure S10), most of which were planted between the 19th and 20th century (Table S1). Although this could imply that even Rockforest trees are reintroduced, palynological and macrofossil evidence strongly support an autochthonous origin of Rockforest pine [30, 33, 39]. Interestingly, at *K* ≥ 5 Rockforest was inferred to harbour genotypic proportions which distinguish it from all other Irish populations (Fig. 8; Figure S12). This differentiation is clearly very minor given the *F*_ST_ values for this population are small (Fig. 4). It is therefore unlikely to correspond to any biologically meaningful genetic structure, especially as the overall variation between Irish populations was not great enough for any substructure to be inferred using the spatially explicit clustering approach of TESS3 (Figure S13). However, the subtle shift in allele frequency which accounts for this signal may nonetheless be the extent to which this relict has become genetically distinct given that its isolation from other native pine (i.e., in Scotland) began only relatively recently. It is also worth noting here that any such signal in nDNA variation may become even more difficult to detect in the future with the effects of homogenising gene flow from reintroduced pine.

### Is there a genetic link between Irish and Scottish Scots pine?

The hypothesised contribution of Irish Scots pine to Scottish pinewoods is one of the big outstanding questions regarding Scots pine in Ireland and Britain. Some evidence suggests no contribution. For example, the Scottish mitotype which Sinclair et al. [105] suggested to be of Irish origin was subsequently demonstrated to occur throughout Europe in a geography-independent manner [109], which rules out it being an Irish-specific mitotype. Others have argued that the Irish Sea constituted an impermeable barrier to tree migration, with early establishment of pine in England being enough to preclude any significant contribution of Irish pine to Scottish pinewoods [91, 110].

Previous work on *Quercus* species, with much less mobile seed, suggested two colonisation routes into Ireland, from the south west across the Celtic Sea and from the east across the Irish Sea [111]. More recent genetic evidence also suggests at least some post-LGM migration from Britain into Ireland for downy birch (*Betula pubescens* Ehrh.) [47] and common ash (*Fraxinus excelsior* L.) [82]. This corroborated the direction of travel which had previously been inferred from isochrone maps based on radiocarbon-dated pollen for both species [21]. These maps suggest an alternative direction of travel for Scots pine from the south-west of Ireland to the north-east and across to Scotland [21]. Indeed, the Irish Sea has recently been redefined as a “filter” rather than a barrier to seed migration [112, 113], which suggests that migration into Scotland could have occurred. Moreover, wind dispersal of Scots pine pollen is highly efficient, with the potential to travel hundreds of kilometres from southern to northern locations [114, 115]. It is possible that pollen from northern Irish populations could reach Scottish populations, given the prevailing southwest wind direction between Ireland and Scotland, coupled with the smallest distance between both being less than 20 km. Indeed, over millennial timescales, a rare event becomes almost inevitable.

Other works have also alluded to a south-western origin for western Scottish pine. For example, variation at nSSR, isozyme and monoterpene loci in western Scottish pine is more similar to southern rather than northern and central European pine [34, 98, 116]. The absence of any strong differentiation between Rockforest and reintroduced Scottish Scots pine could potentially be attributable to both originating from the same gene pool. The plantation stock may therefore derive from native Scottish pine which originally migrated from Ireland following the LGM. Resolving this question using molecular markers will be challenging due to the lack of differentiation in Scots pine and uncertainty regarding other suitable sites to test in Ireland. Further insights may be gleaned by sequencing the mitochondrial genome of Rockforest and western Scottish Scots pine, or even sequencing of DNA from preserved pine stumps or fossil pollen elsewhere in Ireland to identify genotypes across a more extended time period. For example, Danusevičius et al. [117] were able to demonstrate an early presence of mitotype *b* in Lithuania based on DNA extracted from 11,000 year-old Scots pine stumps found in the Baltic Sea, while Bennett and Parducci [118] sequenced DNA from 10,000 year-old fossil pollen grains of Scots pine retrieved from lake sediment in Sweden.

## Conclusions

Whilst Scots pine underwent a regional decline during the Holocene, it is probable that efficient gene flow and a high pre-existing diversity buffered against the species’ terminal decline in Ireland. The results presented here shed some light on the complicated history of Scots pine in Ireland and suggest agreement with the thesis that Irish and Scottish Scots pine share their origins. The lack of clear genetic structure between a putative native Irish population and other populations reintroduced from Scotland suggests that the species across Ireland and Britain exists as a meta-population. This is further underscored by their differentiation from three continental populations and three Norwegian genotypes at nSSR loci. However, more extensive comparison of native Scottish to Irish populations will be needed to confirm this. Whilst it may signal autochthony, the slight shift in allele frequency in Rockforest pine would not seem enough for this population to be treated as genetically distinct from reintroduced pine. Therefore, all Scots pine in Ireland should be managed as if native, as recommended from previous ecological work [8, 39]. Only one population (Derrybawn – Glendalough) harboured trees which appear to be of continental origin, although this was mixed. These results are the first to describe the genetic diversity of a native Irish conifer in a European context and complement recent descriptions of the same for several broadleaf species [47, 82, 119, 120]. This forms part of an inventory of Ireland’s forest genetic resources [121], which is an important step in determining population selection for both in situ and *ex situ* forest conservation approaches [122].

### Supplementary Information

**Additional file 1.** This file contains scripts used for analysing the data

** Additional file 2: Table S1. **Population names, locations, origins and year of planting, where known, along with haplotype designations and number of individuals per population.

**Additional file 3: Table S2. **Chloroplast microsatellite (cpSSR) primers used to genotype *Pinus sylvestris* individuals in this work. **Table S3. **Nuclear microsatellite (nSSR) primers used to genotype *Pinus sylvestris* individuals in this work. **Table S4a. **Results of SAMOVA analysis, unconstrained by geographic data. **Table S4b. **Results of SAMOVA analysis, constrained by geographic data. **Table S5. **Estimates of haplotype genetic diversity (*H*_CP_) and expected heterozygosity (*H*_e_) based on chloroplast (cpSSR) and nuclear (nSSR) SSR genotyping of *Pinus sylvestris* populations from various regions across its distribution range. Mean values are ± S.D. **Table S6. **Estimates of *H*_CP_ based on cpSSR loci variation in *Pinus sylvestris*. In each case, *H*_e_ for Irish trees sampled in this work have been re-estimated according to the loci subsets indicated for different works. **Figure S1. **Mean Nei’s standard and Bruvo’s genetic distance between Scots pine individuals genotyped at cpSSR markers in each sampling population. Error bars are ± S.E. **Figure S2. **Pairwise F_ST_ comparisons between sampled populations. Estimates are based on variation in cpSSR allelic frequency according to Nei (1987). Values which are significantly different from zero are marked with asterisks (*p ≤ 0.05; **p ≤ 0.01; p*** ≤ 0.001; *n* = 348). **Figure S3. **Genetic diversity (*H*_e_) and allelic richness (rarefaction down to 14 nSSR alleles) for each population. Error bars are 95% CIs derived from 1,000 bootstrap permutations of the data. Estimates in this case were calculated after removing the size loci (PtTX3116, SPAC11_6, psy117, PtTX3107, psy12 and psy125) displaying null allele frequencies above 10%. **Figure S4. **Pairwise *R*_ST_ comparisons between sampled populations. Estimates are based on variation of nSSR allele size differences. Values which are significantly different from zero are marked with asterisks (*p ≤ 0.05; **p ≤ 0.01; p*** ≤ 0.001; *n* = 344). **Figure S5. **Mean (a) Nei’s standard and (b) Bruvo’s genetic distance between Scots pine individuals genotyped at nSSR markers in each sampling population. Error bars are ± S.E. **Figure S6. **Distribution of *r *estimated under maximum likelihood using nSSR allele variation in sampled Scots pine (*Pinus sylvestris*) trees. **Figure S7. **Distribution of r estimated under maximum likelihood for each population (including the three and four genotypes from Norway and Scotland (Scottish (Coillte) were samples from a breeding population), respectively) based on nSSR variation in sampled Scots pine (*Pinus sylvestris*) trees. **Figure S8. **Frequency of different pedigree relationships inferred under ML for each population (including the three and four genotypes from Norway and Scotland (Scottish (Coillte)), respectively) based on nSSR variation in sampled Scots pine (*Pinus sylvestris*) trees. **Figure S9.** Principal Coordinates Analysis (PCoA) of the cpSSR allelic composition of each Scots pine (*Pinus sylvestris*) population. Shown are the first and second principal coordinates. Labels are coloured according to population separation in the SAMOVA analysis at *K*=2. Coloured shapes are also drawn around these points to assist in interpretation. **Figure S10. **Results of STRUCTURE analysis of Scots pine (*Pinus sylvestris*) nSSR data. In this case, six loci (PtTX3116, SPAC11_6, psy117, PtTX3107, psy12 and psy125) displaying null allele frequencies above 10% were removed prior to STRUCTURE runs. (a) (i) Mean posterior probability values of *K* (L(*K*)), error bars are ± S.D. (ii) Delta *K* values used to infer the main structure of the data. (b) Frequency of *K* groups within each sampling population including those located in Spain, France and Norway (inset). (c) Bar plots of admixture coefficients for each sampled individual. **Figure S11. **Principal Coordinates Analysis (PCoA) of the nSSR allelic composition of each Scots pine (*Pinus sylvestris*) population. Shown are the first and second principal coordinates. Labels are coloured according to the null allele-adjusted STRUCTURE group (*K*=2) which is most frequent in each population. Coloured shapes are also drawn around these points to assist in interpretation. Loci (PtTX3116, SPAC11_6, psy117, PtTX3107, psy12 and psy125) which displayed null allele frequencies above 10% were removed prior to analysis. **Figure S13. **Results of an analysis of genotype groups (*K*) using TESS3. (a) Given are the cross-validation (CV) scores based on the root-mean squared errors between the genotypic frequencies predicted from a training set to those computed from a test set (i.e., genotype likelihood) for each locus. (b) The frequency of *K* groups within each population are given, including (inset) populations from Spain, France and Norway (for Norway only three individual are used). (c) Individual *K* ancestral proportions for each individual. **Figure S14. **Estimates of expected heterozygosity (*H*_e_) for *Pinus sylvestris* from three different regions based on 18 nSSR loci (grey) and a reduced set of 12 loci (white). The latter remained after removing six loci (PtTX3116, SPAC11_6, psy117, PtTX3107, psy12 and psy12) which displayed null allele frequencies above 10%. Error bars are 95% CI values derived from 1,000 bootstrap permutations of the data. 

**Additional file 4: Table S7.** Raw cpSSR and nSSR data.

** Additional file 5: Figure S12. **Individual bar plots showing admixture coefficients for *K *= 2 to *K *= 15 ancestral groups derived from a STRUCTURE analysis of Scots pine (*Pinus sylvestris*) nSSR variation. Prior to the analysis, six loci (PtTX3116, SPAC11_6, psy117, PtTX3107, psy12 and psy125) displaying null allele frequencies above 10% were removed.

## Data Availability

The raw cpSSR and nSSR data are available in Table S7 for use in future works.
