# Supplementary material for: Molecular characterisation of Pinus sylvestris (L.) in Ireland at the western limit of the species distribution
Source: BMC Ecol Evol. 2024 Jan 23;24:12. doi: 10.1186/s12862-023-02181-3 (PMC10807061; doi:10.1186/s12862-023-02181-3)
Supplement: Supplementary file 1 — Additional file 1. This file contains scripts used for analysing the data [file 12862_2023_2181_MOESM1_ESM.pdf]

Additional file 1. This file contains scripts used for analysing the data.

```
#This script is for extracting haplotypes from the supplied pine cpSSR
data and calculating private haplotype frequency
#To get the input data, tab one in the supplementary file "Table
S7_raw_SSR_data.xlsx" should be saved as a .csv file (e.g.,
"pine_cpSSR.csv") in the user's working directory

#packages used
packages <- c("tidyr", "adegenet")
#install if not not already done so
for (i in packages){
  if(! i %in% installed.packages()){
    install.packages(i, dependencies = TRUE)
  }
  require(i)
}

rm(list=ls()) #clear environment
setwd("") ##paste working directory!##
c <- read.csv("pine_cpSSR.csv", row.names = 1, check.names = FALSE) #read
allelic data frame, with individual IDs as row names

c1 <- c[complete.cases(c), ] #remove rows with NA data, if any
c1 <- subset(c1, select = -c(Country)) #remove country variable

l1 <- length(c1) #upper limit to row names to be considered for haplotype
calling

N.haplotypes <- data.frame(unique( c1[, 4:l1] )) #lists haplotypes based
on cpSSR loci
N.haplotypes #haplotype list
nh <- nrow(N.haplotypes) #nh = number of haplotypes
haps <- c(1:nh) #h = vector of 1:nh
N.haplotypes$Haplotype <- haps #adds Haplotype names to haplotype list
N.haplotypes #new haplotype list

library(tidyr)
a <- colnames(c1) #creates vector of column names
a <- a[-(1:3)] #reduces this vector to loci names only
N.haplotypes <- N.haplotypes %>% unite(Hap_combined, a, sep = " ")
#merges loci per haplotype into one identifier called "Hap_combined"
N.haplotypes <- N.haplotypes[,c(2,1)]
N.haplotypes #list of haplotypes and their allelic composition
("Hap_combined")


library(adegenet)
c_G <- c[complete.cases(c), ] #remove rows with NA data, if any
c_G.pop <- c_G$Pop #vector of population identifiers
c_G <- subset(c_G, select = -c(Country, Pop, Longitude, Latitude))
#leaves loci columns only
#Create genind object
c1.G <- df2genind(c_G,
                  NA.char = "NA",
                  ploidy = 1,
                  type = "codom",
```

```
                    pop = c_G.pop)
c1.G #genind object containing allelic data with population identifiers

s <- seppop(c1.G) #list of genind objects, one per population
s <- lapply(s, function(i) genind2df(i)) #convert to list of data frames
s <- lapply(s, function(i) i %>% unite(Hap_combined, a, sep = " ")) #adds
haplotype identifier
s <- lapply(s, function(i) merge(i, N.haplotypes, by = "Hap_combined"))
#adds haplotype ID
s <- lapply(s, function(i) unlist(i$Haplotype)) #leaves only the
haplotype ID
s <- lapply(s, function(i) unique(i)) #keeps only unique haplotypes in
each population

#the below commands produce a data frame which gives the number of
private alleles for each population
s <- data.frame(unlist(s))
colnames(s) <- "Haplotype"
s$pop <- rownames(s)
rownames(s) <- 1:nrow(s)
s <- s[,c(2,1)]
s <- s[! s$Haplotype %in% unique(s[duplicated(s$Haplotype),
"Haplotype"]), ]
s$pop <- gsub('[[:digit:]]+', '', s$pop)
s <- data.frame(table(s$pop))
colnames(s) <- c("Pop", "pa.H")
#write.csv(s, "pa.haplotypes.csv")

#the below commands produce a data frame which gives the frequency of
private alleles (i.e., the number of private alleles per population
relative to the total number of alleles per population)
popn <- data.frame(table(pop(c1.G)))
colnames(popn) <- c("Pop", "n")
pa <- merge(popn, s, by = "Pop", all.x = T)
pa$pa.H_freq <- pa$pa.H/pa$n
pa <- pa[order(pa$pa.H_freq, decreasing = TRUE),]
pa[is.na(pa)] <- 0
pa
write.csv(pa, "pa.haplotypes.csv")
```
