# Supplementary material for: Molecular characterisation of Pinus sylvestris (L.) in Ireland at the western limit of the species distribution
Source: BMC Ecol Evol. 2024 Jan 23;24:12. doi: 10.1186/s12862-023-02181-3 (PMC10807061; doi:10.1186/s12862-023-02181-3)
Supplement: Supplementary file 3 — Additional file 3: Table S2. Chloroplast microsatellite (cpSSR) primers used to genotype Pinus sylvestris individuals in this work. Table S3. Nuclear microsatellite (nSSR) primers used to genotype Pinus sylvestris individuals in this work. Table S4a. Results of SAMOVA analysis, unconstrained by geographic data. Table S4b. Results of SAMOVA analysis, constrained by geographic data. Table S5. Estimates of haplotype genetic diversity (HCP) and expected heterozygosity (He) based on chloroplast (cpSSR) and nuclear (nSSR) SSR genotyping of Pinus sylvestris populations from various regions across its distribution range. Mean values are ± S.D. Table S6. Estimates of HCP based on cpSSR loci variation in Pinus sylvestris. In each case, He for Irish trees sampled in this work have been re-estimated according to the loci subsets indicated for different works. Figure S1. Mean Nei’s standard and Bruvo’s genetic distance between Scots pine individuals genotyped at cpSSR markers in each sampling population. Error bars are ± S.E. Figure S2. Pairwise FST comparisons between sampled populations. Estimates are based on variation in cpSSR allelic frequency according to Nei (1987). Values which are significantly different from zero are marked with asterisks (*p ≤ 0.05; **p ≤ 0.01; p*** ≤ 0.001; n = 348). Figure S3. Genetic diversity (He) and allelic richness (rarefaction down to 14 nSSR alleles) for each population. Error bars are 95% CIs derived from 1,000 bootstrap permutations of the data. Estimates in this case were calculated after removing the size loci (PtTX3116, SPAC11_6, psy117, PtTX3107, psy12 and psy125) displaying null allele frequencies above 10%. Figure S4. Pairwise RST comparisons between sampled populations. Estimates are based on variation of nSSR allele size differences. Values which are significantly different from zero are marked with asterisks (*p ≤ 0.05; **p ≤ 0.01; p*** ≤ 0.001; n = 344). Figure S5. Mean (a) Nei’s standard and (b) Bruvo’s genetic distance between S [file 12862_2023_2181_MOESM3_ESM.docx]

**Table S1.** Population names, locations, origins and year of planting, where known, along with haplotype designations and number of individuals per population.

*See separate file.*

**Table S2.** Chloroplast microsatellite (cpSSR) primers used to genotype Pinus sylvestris individuals in this work.

| **Name** | **Dye** | **PCR plex** | **Expected size** | **Conc. of each primer (µM)** |
| --- | --- | --- | --- | --- |
| Pt26081 | PET | A | 109/112 | 0.4 |
| Pt30204 | 6-FAM | A | 140/145 | 0.5 |
| Pt71936 | VIC | A | 146/148 | 0.5 |
| Pt87268 | NED | A | 165/167 | 0.5 |
| Pt1254 | VIC | B | 72/74 | 0.3 |
| Pt41093 | 6-FAM | B | 78 | 0.3 |
| Pt15169 | NED | B | 115/118 | 0.4 |
| Pt36480 | PET | B | 145/147 | 0.5 |

**Table S3.** Nuclear microsatellite (nSSR) primers used to genotype Pinus sylvestris individuals in this work.

| **Name** | **Dye** | **PCR plex** | **Multiplex for gel migration** | **Expected size** | **Conc. of each primer (µM)** |
| --- | --- | --- | --- | --- | --- |
| SPAC11.6 | PET | 2 | A | 103-220 | 1.2 |
| SPAC12.5 | NED | 2 | A | 116-202 | 1.2 |
| SPAG7.14 | VIC | 2 | A | 174-252 | 1.4 |
| psy12 | 6-FAM | 2 | A | 199-211 | 1.4 |
| PtTX3116 | VIC | 1 | B | 100-276 | 1.2 |
| PtTX3107 | 6-FAM | 1 | B | 144-175 | 1.2 |
| PtTX4001 | NED | 1 | B | 197-231 | 1.4 |
| PtTX4011 | 6-FAM | 1 | B | 230-284 | 1.4 |
| PtTX3032 | PET | 1 | B | 254-572 | 1.4 |
| SPAC11.4 | PET | 4 | C | 130-170 | 1.8 |
| psy144 | VIC | 4 | C | 166-175 | 1.1 |
| psy142 | 6-FAM | 4 | C | 171-179 | 1.8 |
| psy157 | NED | 4 | C | 187-202 | 1.2 |
| psy125 | 6-FAM | 4 | C | 214-244 | 1.8 |
| psy117 | VIC | 3 | C | 219-251 | 1.1 |
| psy136 | NED | 3 | C | 245-257 | 1.2 |
| psy118 | VIC | 3 | C | 297-306 | 0.6 |
| psy119 | NED | 3 | C | 315-324 | 2.0 |

**Additional File 1** – see separate file.

**Table S4a.** Results of SAMOVA analysis, unconstrained by geographic data.

| **K** | **Structure** | **Among groups** | | | **Among populations within groups** | | | **Within populations** | | |
| --- | --- | --- | --- | --- | --- | --- | --- | --- | --- | --- |
|  |  | **Variation (%)** | **F_CT_** | **P-value** | **Variation (%)** | **F_SC_** | **P-value** | **Variation (%)** | **F_ST_** | **P-value** |
| **2** | **Group 1:** Dale Wood, **Group 2:** all others | 8.28 | 0.0828 | 0.0459 | 1.29 | 0.0141 | 0.0802 | 90.43 | 0.0957 | 0.0147 |
| **3** | **Group 1:** Dale Wood, **Group 2:** Glenfarne, **Group 3:** all others | 7.69 | 0.0770 | 0.0059 | 0.8000 | 0.0087 | 0.1672 | 91.5000 | 0.0850 | 0.0127 |
| **4** | **Group 1:** Dale Wood + Torc, **Group 2:** Glenfarne, **Group 3:** Knockastackeen, **Group 4:** all others | 7.27 | 0.0727 | 0.0000 | 0.1100 | 0.0011 | 0.4458 | 92.6200 | 0.0738 | 0.0176 |
| **5** | **Group 1:** Dale Wood + Torc, **Group 2:** Glenfarne, **Group 3:** Knockastackeen, **Group 4:** Cavan, **Group 5:** all others | 6.82 | 0.0682 | 0.0000 | -0.2000 | -0.0022 | 0.5269 | 93.3800 | 0.0662 | 0.0244 |
| **6** | **Group 1:** Dale Wood + Torc, **Group 2:** Glenfarne, **Group 3:** Knockastackeen + Vale of Clara, **Group 4:** Cavan, **Group 5:** Derrycrag Wood, **Group 6:** all others | 6.55 | 0.0655 | 0.0000 | -0.8200 | -0.0088 | 0.7566 | 94.2700 | 0.0573 | 0.0313 |
| **7** | **Group 1:** Dale Wood + Torc, **Group 2:** Glenfarne, **Group 3:** Knockastackeen + Vale of Clara, **Group 4:** Cavan, **Group 5:** Derrycrag Wood, **Group 6:** Glengarriff, **Group 7:** all others | 6.38 | 0.0638 | 0.0000 | -1.1500 | -0.0123 | 0.8632 | 94.7700 | 0.0523 | 0.0196 |
| **8** | **Group 1:** Dale Wood, **Group 2:** Torc, **Group 3:** Glenfarne, **Group 4:** Knockastackeen + Vale of Clara, **Group 5:** Cavan, **Group 6:** Derrycrag Wood, **Group 7:** Glengarriff, **Group 8:** all others | 6.19 | 0.0619 | 0.0000 | -1.0700 | -0.0114 | 0.8201 | 94.8800 | 0.0512 | 0.0156 |
| **9** | **Group 1:** Dale Wood + Derrycrag Wood + Torc, **Group 2:** Breen Wood + Glenfarne, **Group 3:** Glenveagh + Knockastackeen + Sainte-Baume + Vale of Clara, **Group 4:** Cavan + Ballykelly, **Group 5:** Borau + Glengarriff + Rockvale, **Group 6:** Rockforest, **Group 7:** Ballykine, **Group 8:** Trooperstown, **Group 9:** all others | 6.10 | 0.0610 | 0.0000 | -3.5100 | -0.0374 | 0.9990 | 97.4200 | 0.0258 | 0.0127 |
| **10** | **Group 1:** Dale Wood + Torc + Derrycrag Wood, **Group 2:** Breen Wood + Glenfarne, **Group 3:** Knockastackeen + Vale of Clara, **Group 4:** Glenveagh + Sainte-Baume, **Group 5:** Cavan + Ballykelly, **Group 6:** Borau + Glengarriff + Rockvale, **Group 7:** Rockforest, **Group 8:** Ballykine, **Group 9:** Trooperstown, **Group 10:** all others | 6.29 | 0.0629 | 0.0000 | -3.7500 | -0.0401 | 1.0000 | 97.4600 | 0.0254 | 0.0186 |

**Table S4b.** Results of SAMOVA analysis, constrained by geographic data.

| **K** | **Structure** | **Among groups** | | | **Among populations within groups** | | | **Within populations** | | |
| --- | --- | --- | --- | --- | --- | --- | --- | --- | --- | --- |
|  |  | **Variation (%)** | **F_CT_** | **P-value** | **Variation (%)** | **F_SC_** | **P-value** | **Variation (%)** | **F_ST_** | **P-value** |
| **2** | **Group 1:** Dale Wood, **Group 2:** all others | 8.28 | 0.0828 | 0.0489 | 1.2900 | 0.0141 | 0.0860 | 90.4300 | 0.0957 | 0.0225 |
| **3** | **Group 1:** Dale Wood, **Group 2:** Glenfarne, **Group 3:** all others | 7.69 | 0.0770 | 0.0049 | 0.8000 | 0.0087 | 0.1828 | 91.5000 | 0.0850 | 0.0147 |
| **4** | **Group 1:** Dale Wood + Torc, **Group 2:** Glenfarne, **Group 3:** Knockastackeen, **Group 4:** all others | 7.27 | 0.0727 | 0.0000 | 0.1100 | 0.0011 | 0.4575 | 92.6200 | 0.0738 | 0.0108 |
| **5** | **Group 1:** Dale Wood + Torc**, Group 2:** Glenfarne, **Group 3:** Knockastackeen, **Group 4:** Cavan, **Group 5:** all others | 6.82 | 0.0682 | 0.0000 | -0.2000 | -0.0022 | 0.5386 | 93.3800 | 0.0662 | 0.0156 |
| **6** | **Group 1:** Dale Wood + Torc, **Group 2:** Glenfarne, **Group 3:** Knockastackeen + Vale of Clara, **Group 4:** Cavan, **Group 5:** Derrycrag Wood, **Group 6:** all others | 6.55 | 0.0655 | 0.0000 | -0.8200 | -0.0088 | 0.7625 | 94.2700 | 0.0573 | 0.0235 |
| **7** | **Group 1:** Dale Wood + Torc, **Group 2:** Glenfarne, **Group 3:** Knockastackeen + Vale of Clara, **Group 4:** Cavan, **Group 5:** Derrycrag Wood, **Group 6:** Glengarriff, **Group 7:** all others | 6.38 | 0.0638 | 0.0000 | -1.1500 | -0.0123 | 0.8563 | 94.7700 | 0.0523 | 0.0176 |
| **8** | **Group 1:** Dale Wood**, Group 2:** Torc, **Group 3:** Glenfarne, **Group 4:** Knockastackeen + Vale of Clara, **Group 5:** Cavan, **Group 6:** Derrycrag Wood, **Group 7:** Glengarriff, **Group 8:** all others | 6.19 | 0.0619 | 0.0000 | -1.0700 | -0.0114 | 0.8319 | 94.8800 | 0.0512 | 0.0176 |
| **9** | **Group 1**: Dale Wood, **Group 2:** Torc, **Group 3:** Glenfarne, **Group 4:** Knockastackeen + Vale of Clara, **Group 5:** Cavan, **Group 6:** Derrycrag Wood, **Group 7:** Glengarriff, **Group 8:** Sainte-Baume, **Group 9:** all others | 6.00 | 0.0600 | 0.0000 | -1.2900 | -0.0137 | 0.9091 | 95.2900 | 0.0471 | 0.0137 |
| **10** | **Group 1:** Dale Wood + Torc, **Group 2:** Glenfarne, **Group 3:** Knockastackeen + Vale of Clara, **Group 4:** Cavan, **Group 5:** Derrycrag Wood, **Group 6:** Borau + Glengarriff, **Group 7:** Rockforest, **Group 8:** Trooperstown, **Group 9:** Ballykine + Rockvale, **Group 10:** all others | 6.13 | 0.0613 | 0.0000 | -3.4100 | -0.0363 | 1.0000 | 97.2700 | 0.0273 | 0.0205 |

**Table S5.** Estimates of haplotype genetic diversity (H_CP_) and expected heterozygosity (H_e_) based on chloroplast (cpSSR) and nuclear (nSSR) SSR genotyping of Pinus sylvestris populations from various regions across its distribution range. Mean values are ± S.D.

| **cpSSR** | ***H*_CP_** | **Reference** |
| --- | --- | --- |
| Ireland | 0.967 | This work |
| Scotland | 0.982-1 | [1, 2] |
| Spain | 0.763, 0.978, 0.983 | [3, 4], this work |
| Portugal | 0.781 | [3] |
| Northern Italy | 0.92 | [5] |
| France | 0.822-0.910 | [3], this work |
| Poland | 0.986-0.992 | [6, 7] |
| Bulgaria | 0.695-0.984 | [3, 8] |
| Sweden | 0.828 | [3] |
| Finland | 0.752 | [3] |
| Greece | 0.757 | [3] |
| Serbia | 0.571 | [3] |
| Andorra | 0.868 | [3] |
| Turkey | 0.742 | [3] |
| Romanian Carpathians | 0.99 | [9] |
| Crimea, the Lesser Caucasus and Asia Minor | 0.971 | [8] |
| Eastern Caucasus | 0.848 | [8] |
| Western Caucasus | 0.913 | [8] |
| Lithuania | 0.76-0.991 | [7, 10] |
| Estonia | 0.999 | [7] |
| Latvia | 1 | [7] |
| European Russia | 0.984-0.995 | [7, 11] |
| Siberia | 0.976-0.99 | [9, 11] |
| Transbaikalia and Mongolia | 0.967 | [11] |
| mean | 0.888 ± 0.109 | - |
| **nSSR** | ***H_e_*** | **Reference** |
| Ireland | 0.540 | This work |
| Scotland | 0.576-0.76 | [12, 13] |
| Northern Italy | 0.847, 0.81 | [5, 14] |
| Finland | 0.762 | [13] |
| Norway | 0.711 | [13] |
| Sweden | 0.763 | [13] |
| Romania and Hungary (includes plantations) | 0.55 | [15] |
| Lithuania | 0.59-0.78 | [10, 13] |
| Estonia | 0.73 | [13] |
| Eastern Carpathians | 0.678 | [16] |
| Southern Carpathians | 0.729 | [16] |
| Greater Caucasus | 0.588 | [17] |
| Lesser Caucasus | 0.559 | [17] |
| West Anatolia | 0.587 | [17] |
| East Anatolia | 0.587 | [17] |
| Northwest Russia | 0.634 | [18] |
| mean | 0.660 ± 0.091 | - |

**Table S6.** Estimates of H_CP_ based on cpSSR loci variation in Pinus sylvestris. In each case, H_e_ for Irish trees sampled in this work have been re-estimated according to the loci subsets indicated for different works.

| **Region** | ***H*_CP_** | ***H*_CP_ for Ireland** | **Loci used** | **Reference** |
| --- | --- | --- | --- | --- |
| Northern Spain | 0.978 | 0.965 | PtI5169, Pt26081, Pt30204, Pt36480,  Pt71936, Pt8726 | [4] |
| Northern Italy | 0.92 | 0.738 | Pt30204, Pt71936 | [5] |
| Lithuania | 0.76 | 0.943 | PtI5169, Pt30204, Pt71936 | [10] |
| European Russia | 0.984 | 0.943 | Pt15169, Pt26081, Pt30204, Pt71936 | [11] |
| Siberia and Kazakhstan | 0.976 | 0.923 | Pt15169, Pt26081, Pt30204, Pt71936 | [11] |
| Transbaikalia and Mongolia | 0.967 | 0.943 | Pt15169, Pt26081, Pt30204, Pt71936 | [11] |
| Crimea, Caucasus and Asia Minor | 0.898 | 0.943 | Pt15169, Pt26081, Pt30204, Pt71936 | [8] |
| Europe and North Asia | 0.975 | 0.943 | Pt15169, Pt26081, Pt30204, Pt71936 | [8] |

**Table S7**. Raw cpSSR and nSSR data.

*See separate file.*


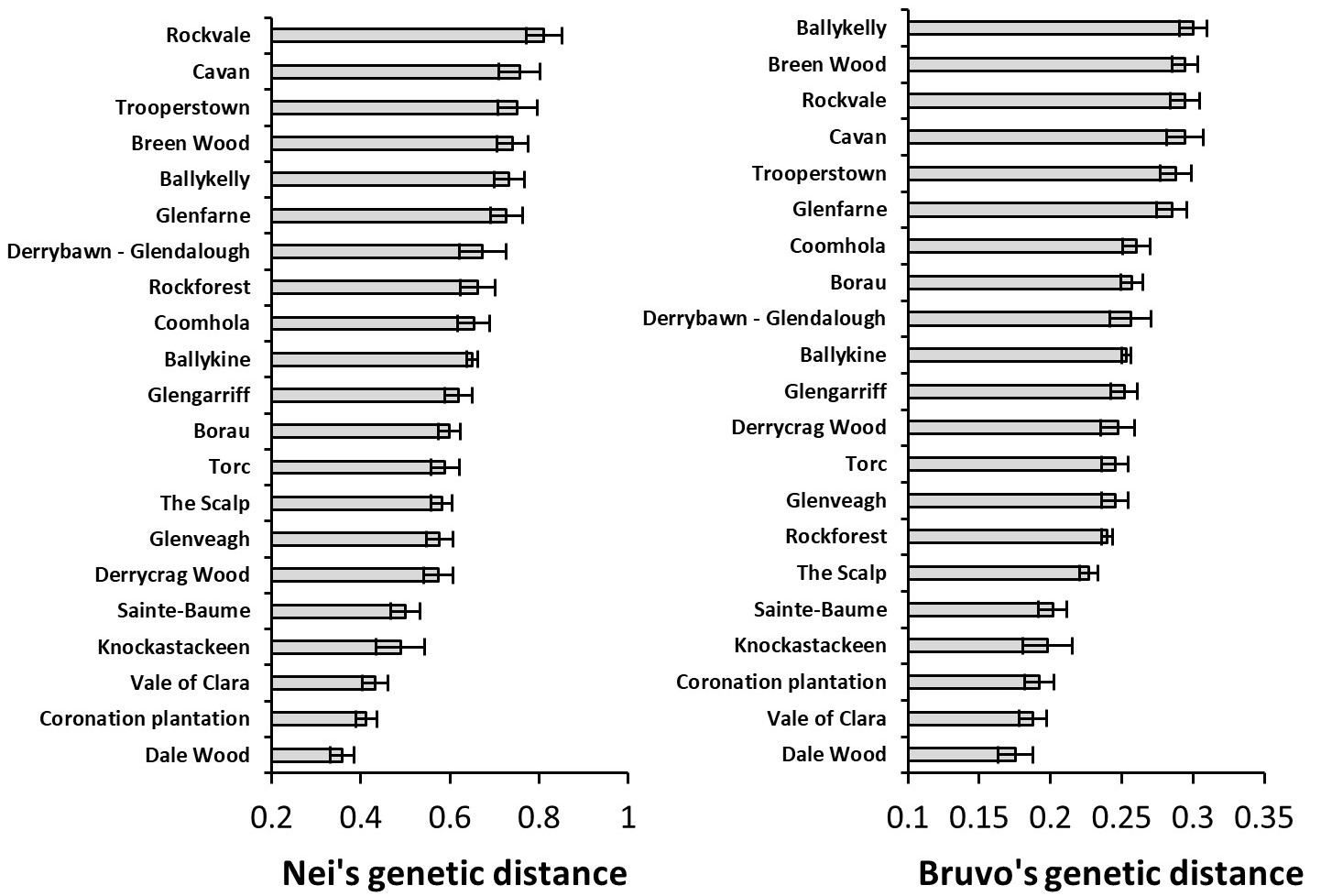


**Figure S1.** Mean Nei’s standard and Bruvo’s genetic distance between Scots pine individuals genotyped at cpSSR markers in each sampling population. Error bars are ± S.E.


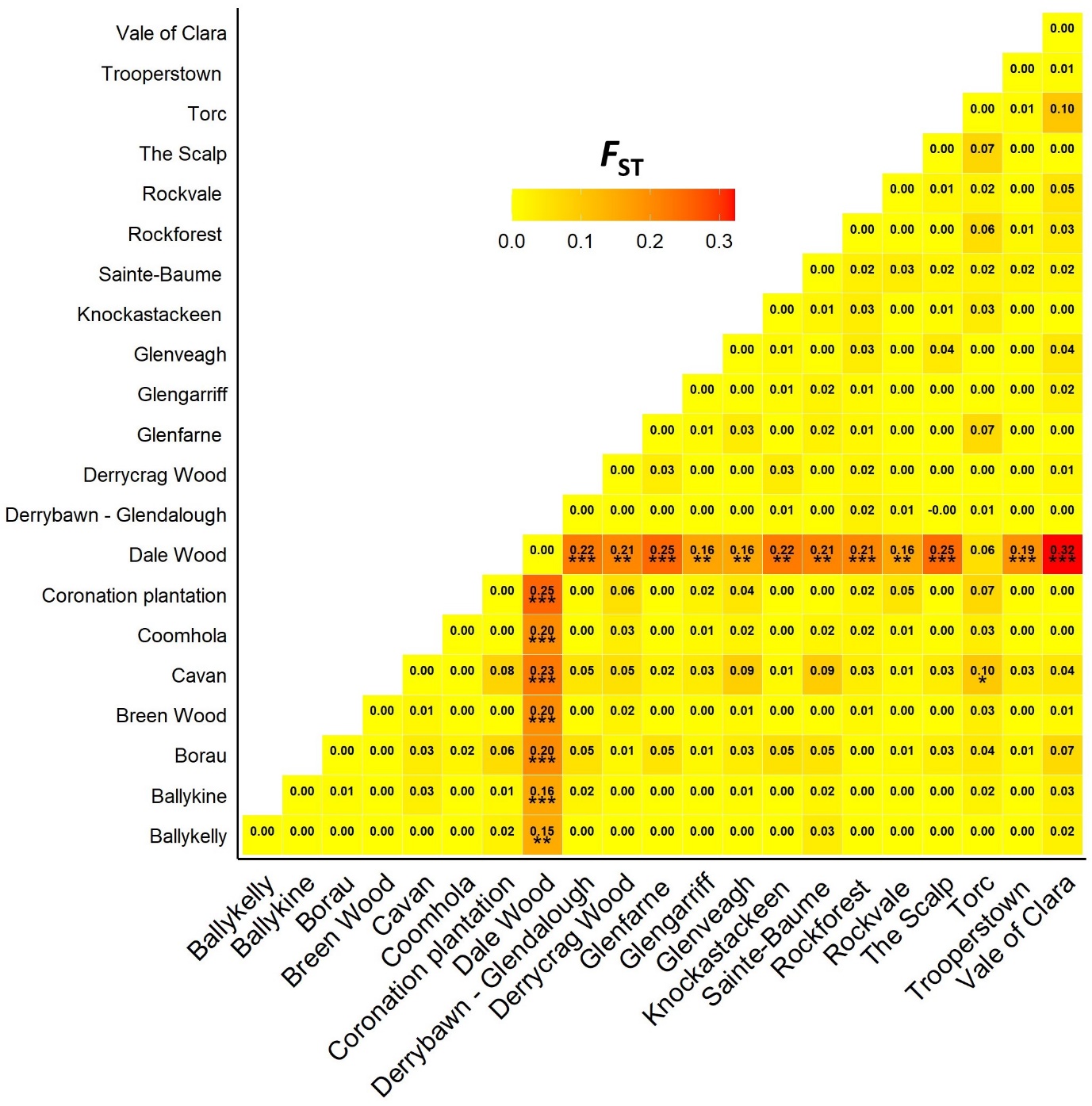


**Figure S2.** Pairwise F_ST_ comparisons between sampled populations. Estimates are based on variation in cpSSR allelic frequency according to Nei (1987). Values which are significantly different from zero are marked with asterisks (*p ≤ 0.05; **p ≤ 0.01; p*** ≤ 0.001; n = 348).


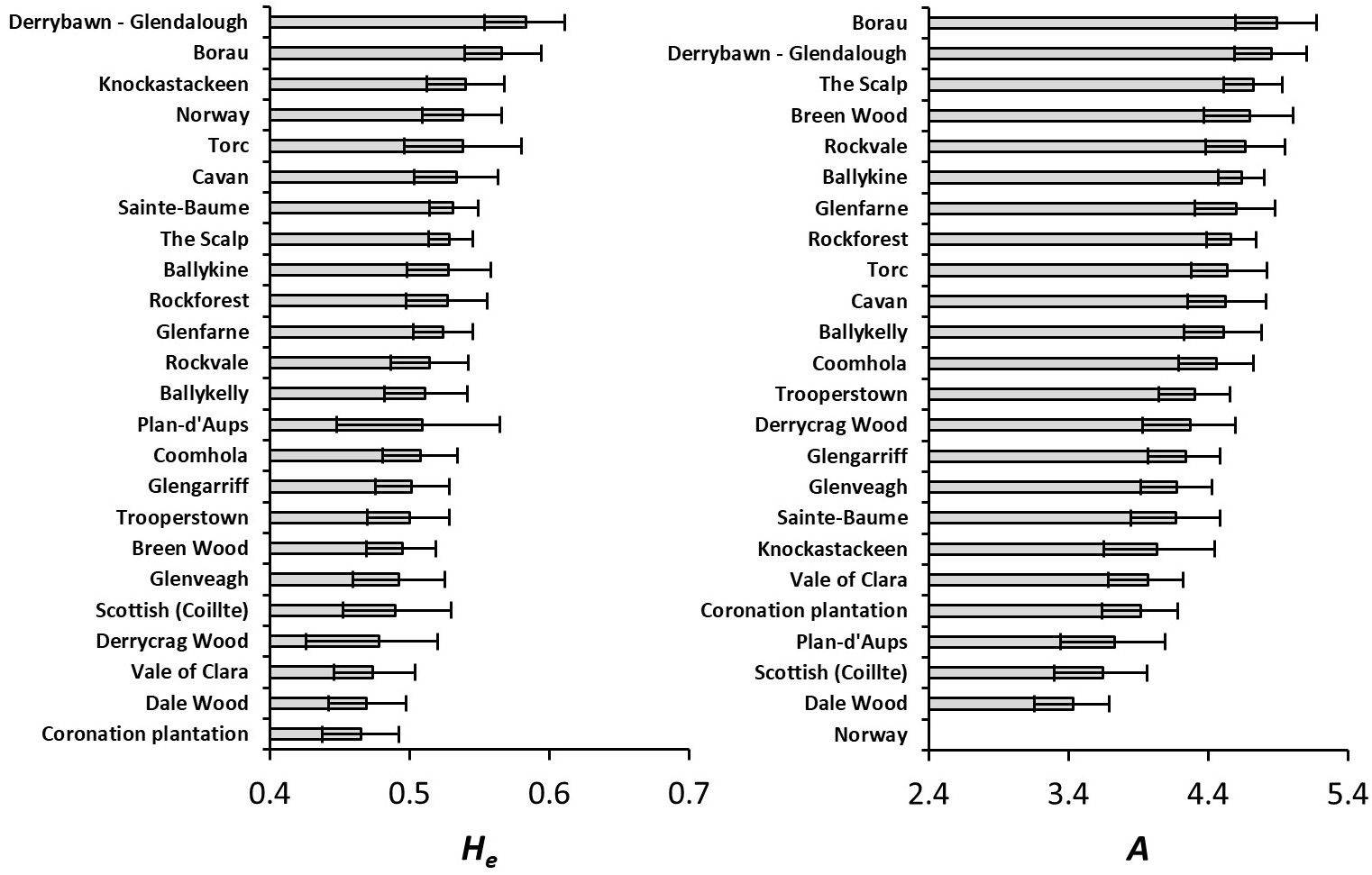


**NA**

**Figure S3.** Genetic diversity (H_e_) and allelic richness (rarefaction down to 14 nSSR alleles) for each population. Error bars are 95% CIs derived from 1,000 bootstrap permutations of the data. Estimates in this case were calculated after removing the size loci (PtTX3116, SPAC11_6, psy117, PtTX3107, psy12 and psy125) displaying null allele frequencies above 10%.


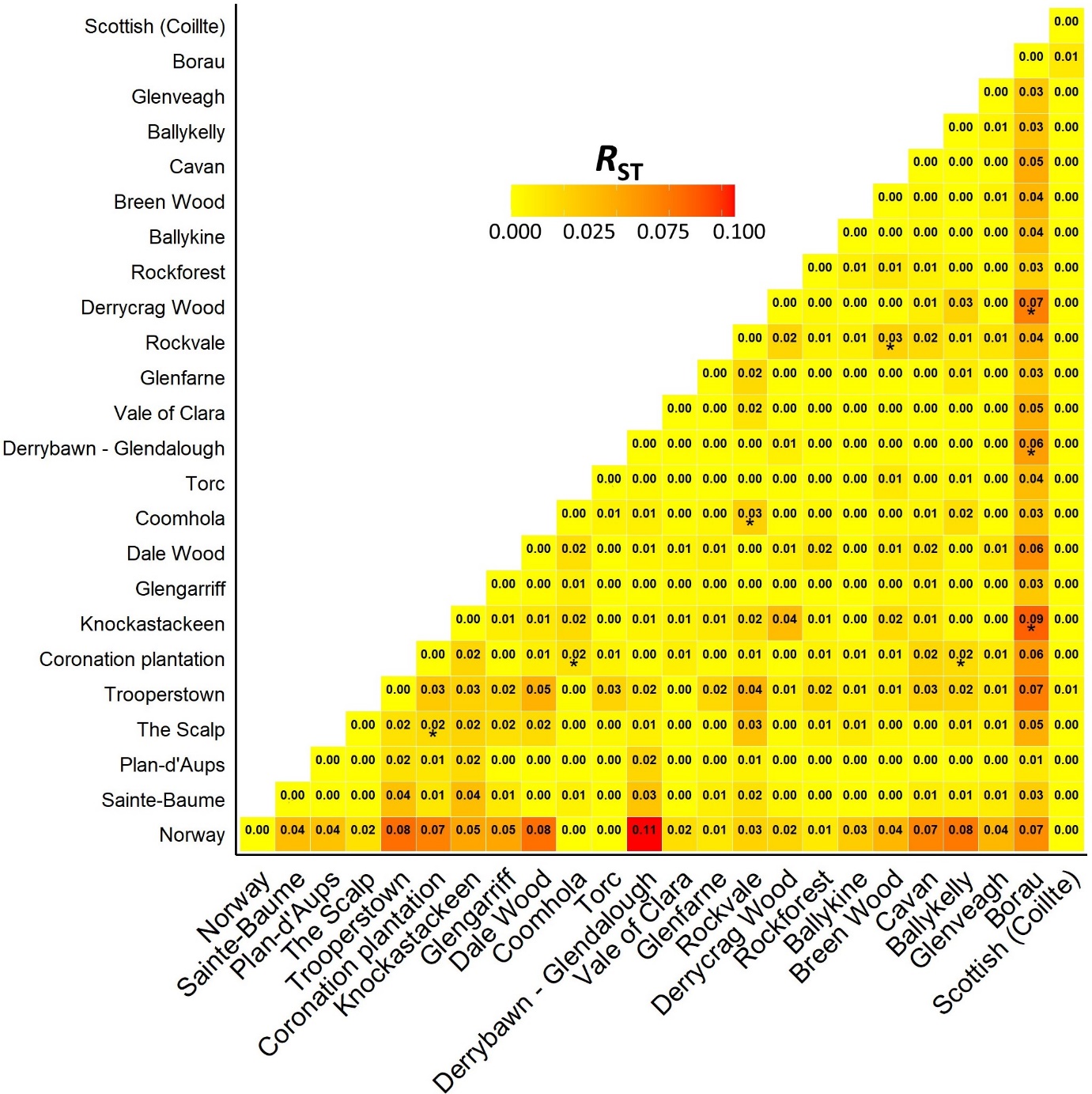


**Figure S4.** Pairwise R_ST_ comparisons between sampled populations. Estimates are based on variation of nSSR allele size differences. Values which are significantly different from zero are marked with asterisks (*p ≤ 0.05; **p ≤ 0.01; p*** ≤ 0.001; n = 344)


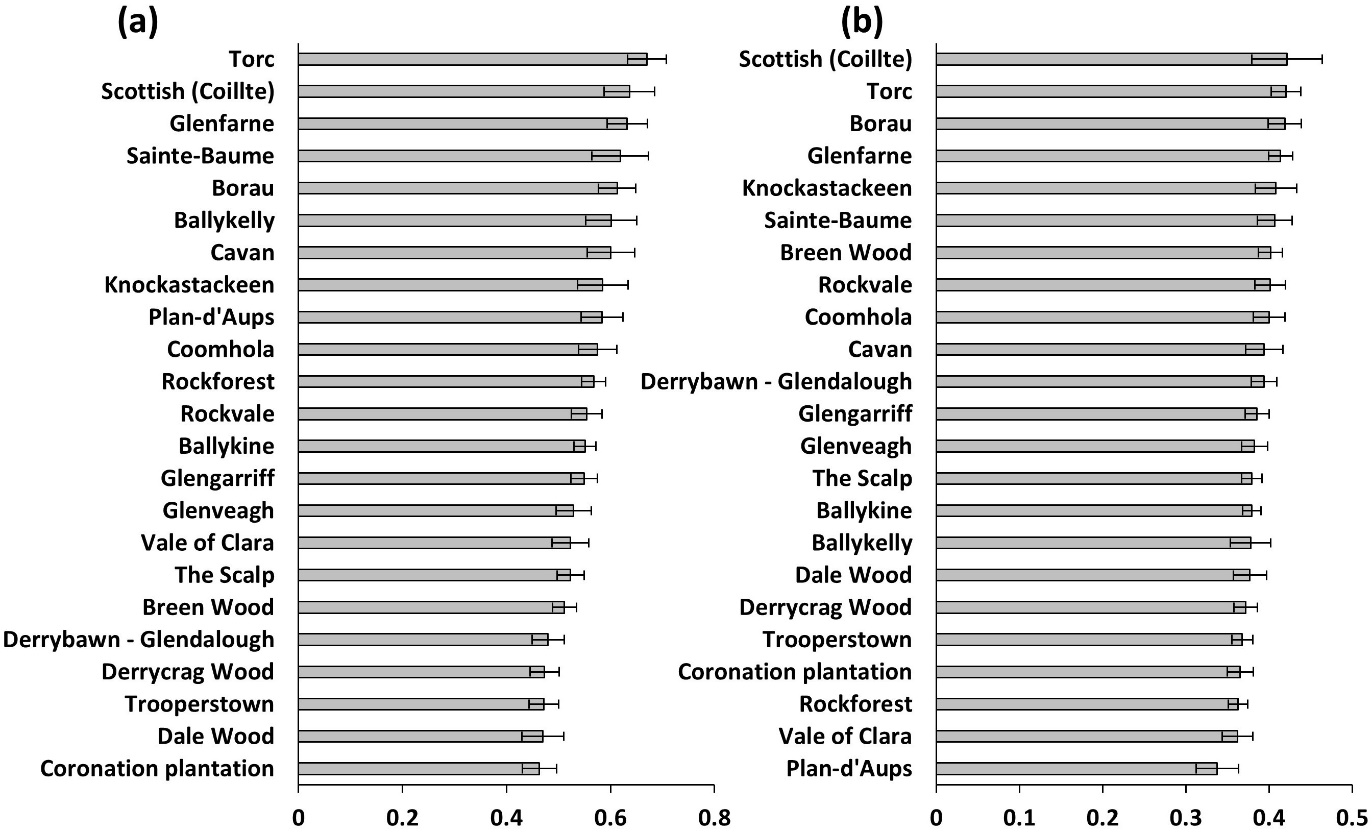


**Figure S5.** Mean **(a)** Nei’s standard and **(b)** Bruvo’s genetic distance between Scots pine individuals genotyped at nSSR markers in each sampling population. Error bars are ± S.E.

**
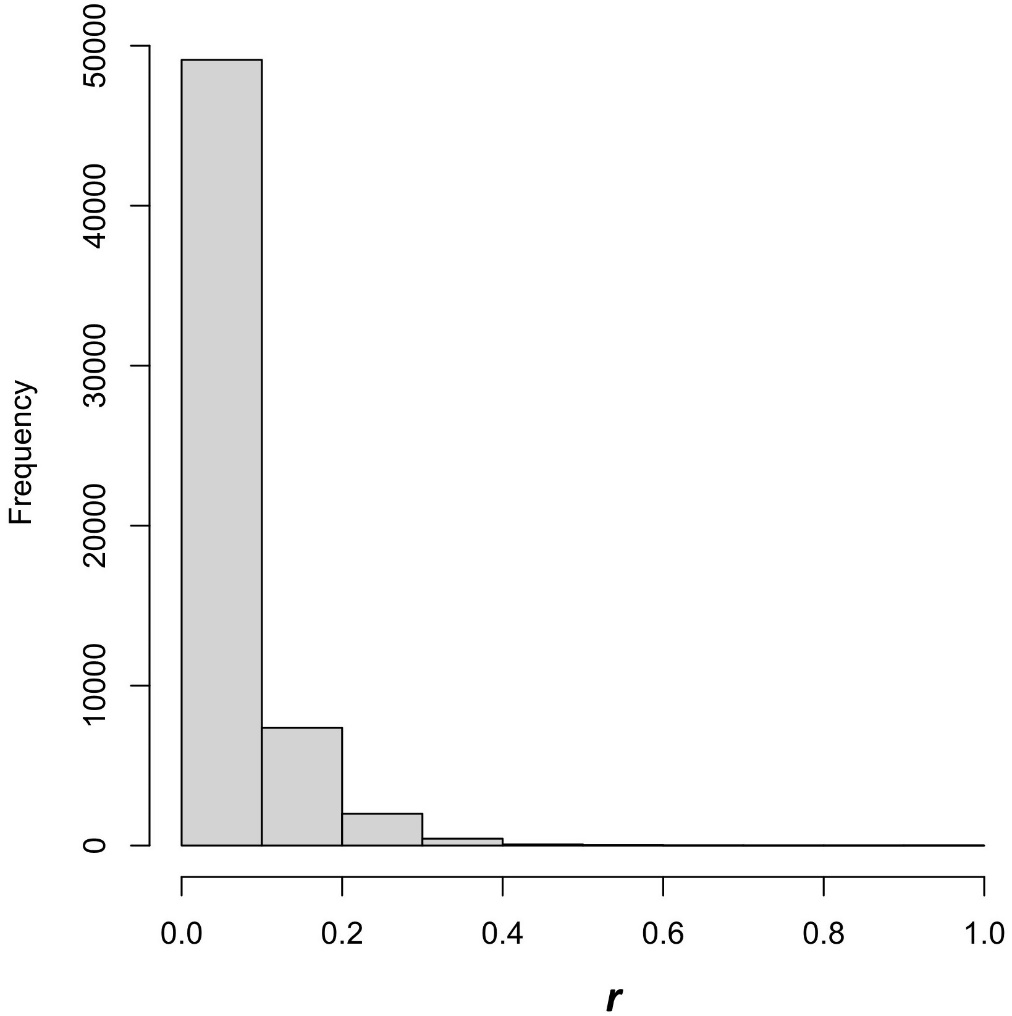
**

**Figure S6.** Distribution of *r* estimated under maximum likelihood using nSSR allele variation in sampled Scots pine (*Pinus sylvestris*) trees.


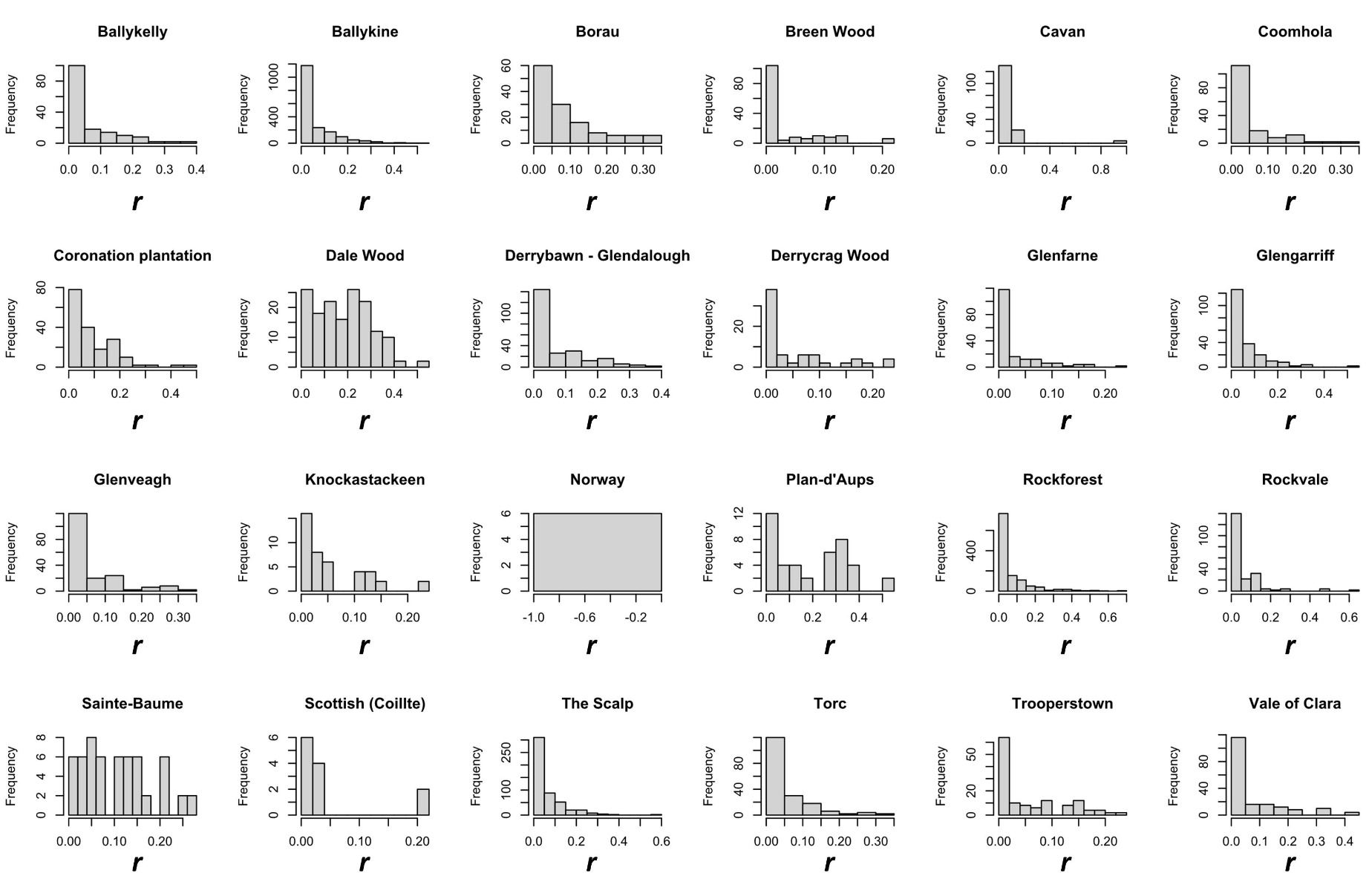


**Figure S7.** Distribution of r estimated under maximum likelihood for each population (including the three and four genotypes from Norway and Scotland (Scottish (Coillte) were samples from a breeding population), respectively) based on nSSR variation in sampled Scots pine (Pinus sylvestris) trees.


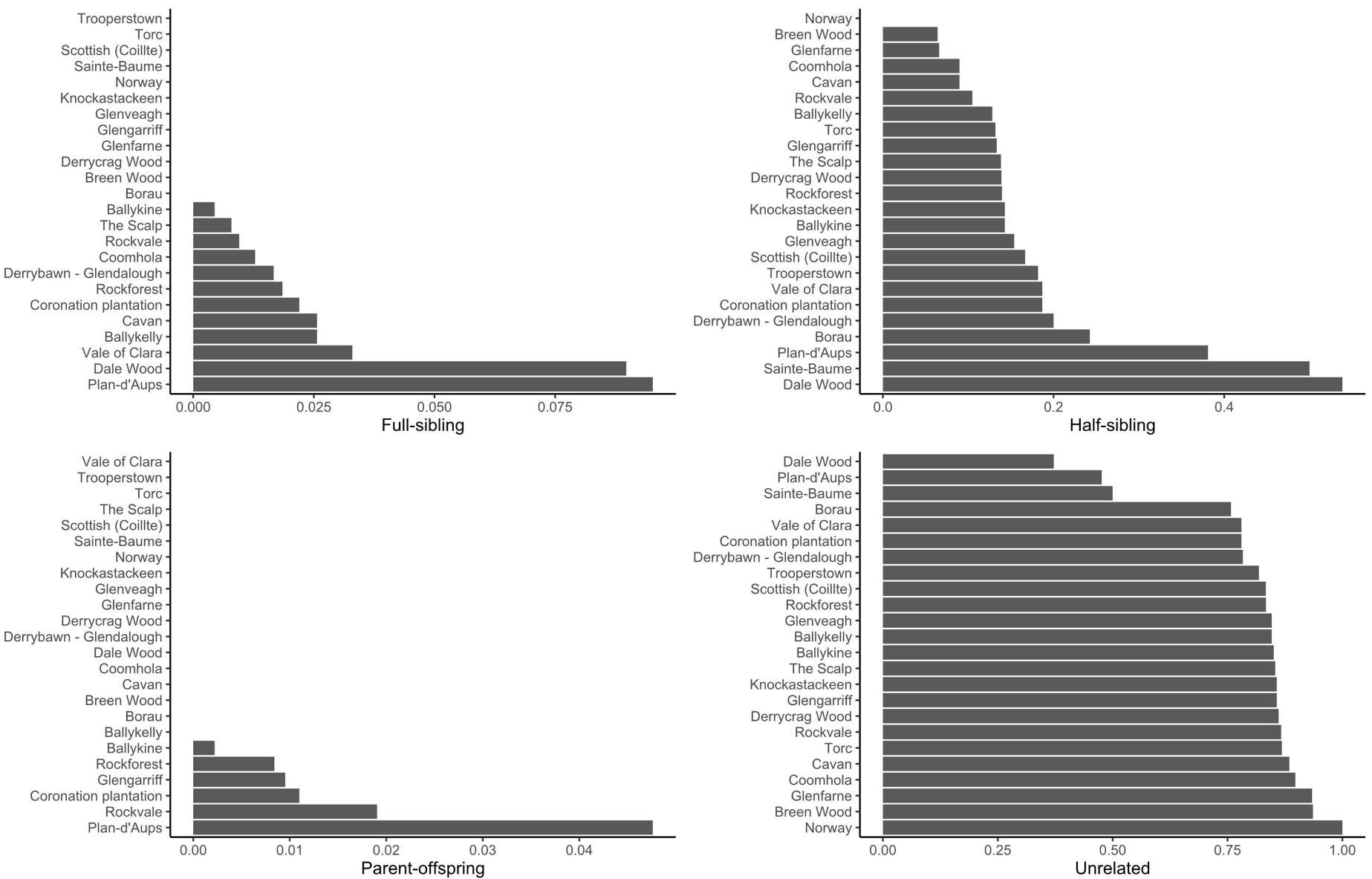


**Figure S8.** Frequency of different pedigree relationships inferred under ML for each population (including the three and four genotypes from Norway and Scotland (Scottish (Coillte)), respectively) based on nSSR variation in sampled Scots pine (Pinus sylvestris) trees.


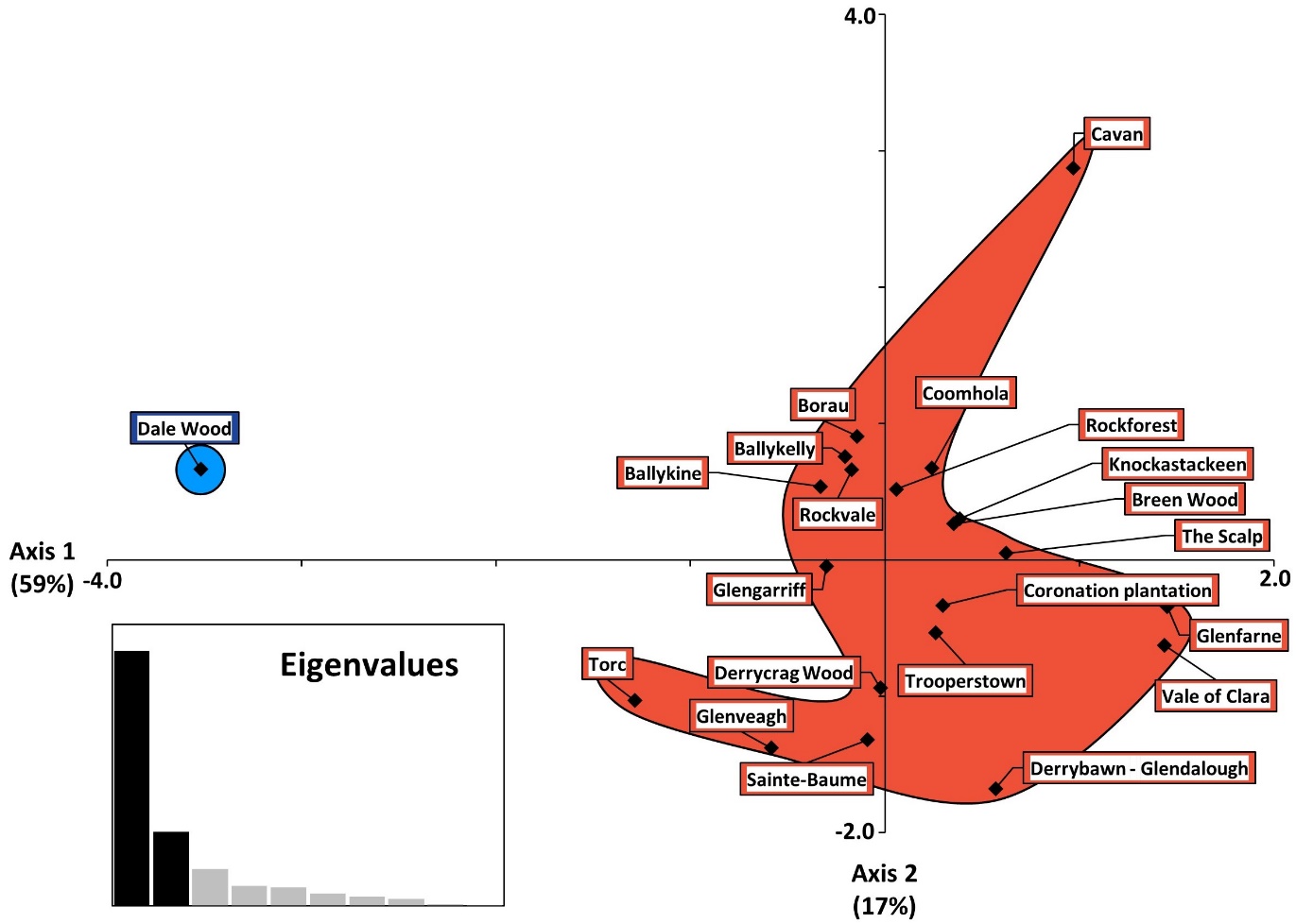


**Figure S9.** Principal Coordinates Analysis (PCoA) of the cpSSR allelic composition of each Scots pine (Pinus sylvestris) population. Shown are the first and second principal coordinates. Labels are coloured according to population separation in the SAMOVA analysis at K=2. Coloured shapes are also drawn around these points to assist in interpretation.


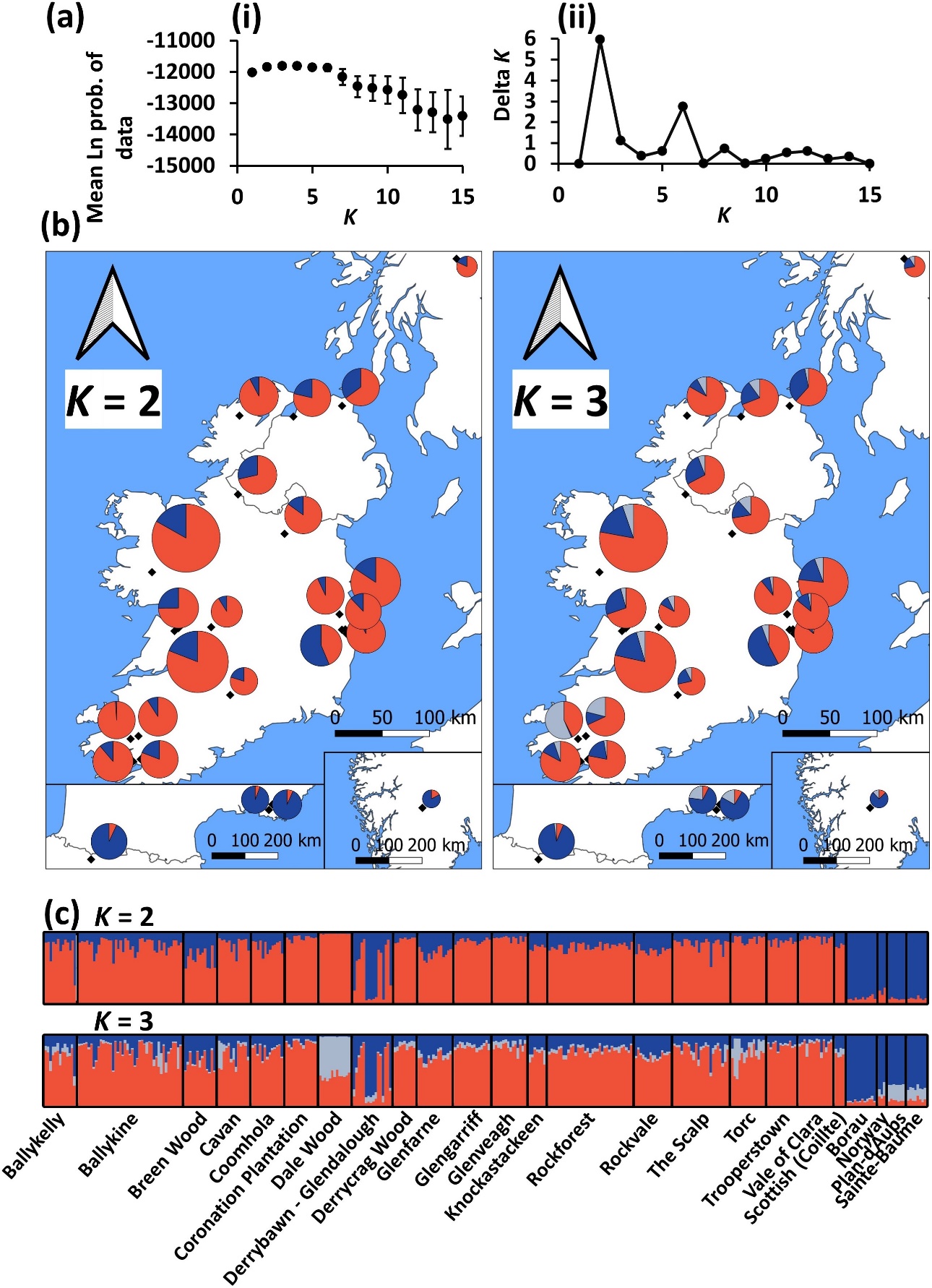


**Figure S10.** Results of STRUCTURE analysis of Scots pine (Pinus sylvestris) nSSR data. In this case, six loci (PtTX3116, SPAC11_6, psy117, PtTX3107, psy12 and psy125) displaying null allele frequencies above 10% were removed prior to STRUCTURE runs. **(a) (i)** Mean posterior probability values of K (L(K)), error bares are ± S.D. **(ii)** Delta K values used to infer the main structure of the data. **(b)** Frequency of K groups within each sampling population including those located in Spain, France and Norway (inset). **(c)** Bar plots of admixture coefficients for each sampled individual.


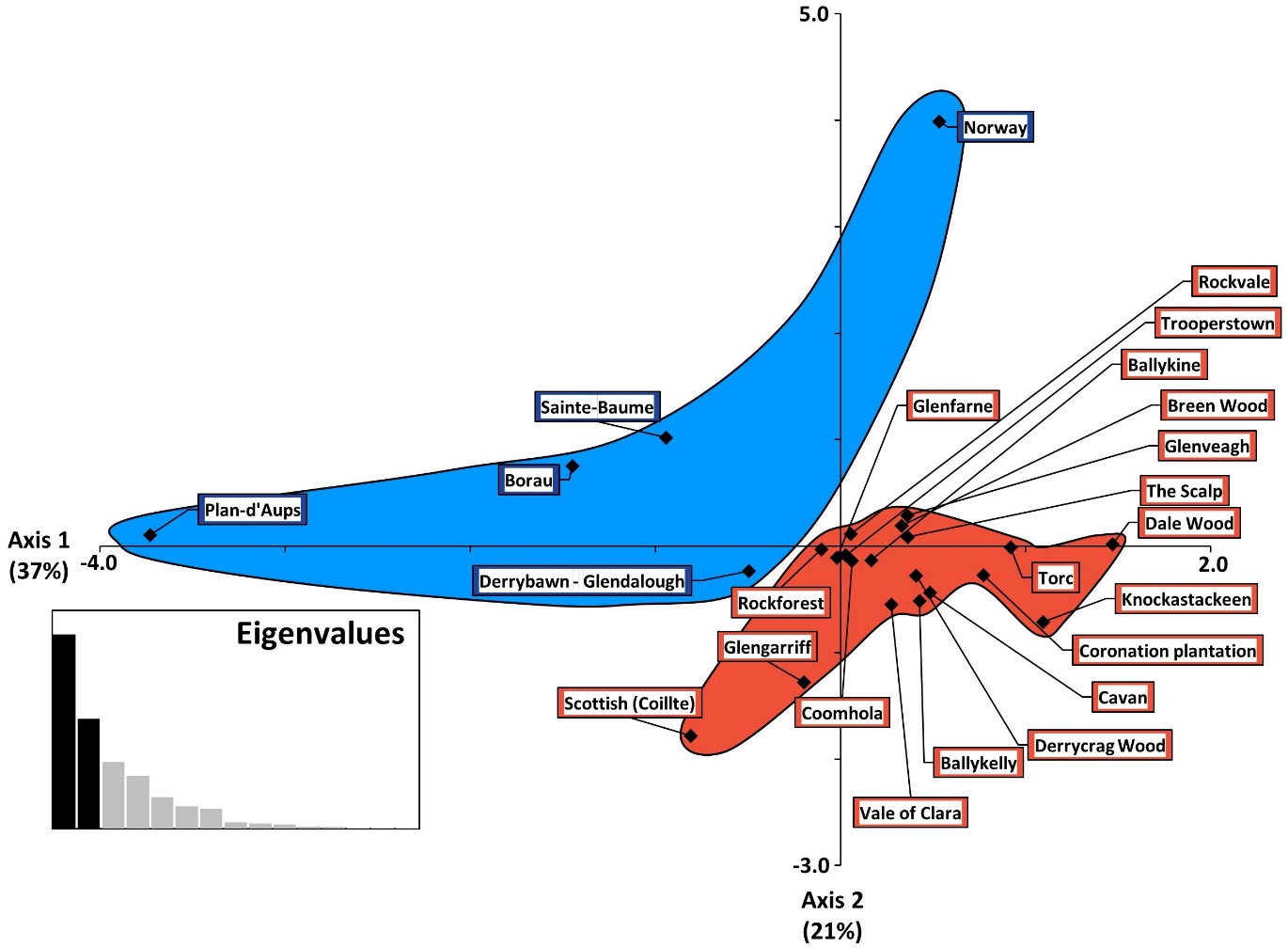


**Figure S11.** Principal Coordinates Analysis (PCoA) of the nSSR allelic composition of each Scots pine (Pinus sylvestris) population. Shown are the first and second principal coordinates. Labels are coloured according to the null allele-adjusted STRUCTURE group (K=2) which is most frequent in each population. Coloured shapes are also drawn around these points to assist in interpretation. Loci (PtTX3116, SPAC11_6, psy117, PtTX3107, psy12 and psy125) which displayed null allele frequencies above 10% were removed prior to analysis.

*See separate file*

**Figure S12.** Individual bar plots showing admixture coefficients for *K* = 2 to *K* = 15 ancestral groups derived from a STRUCTURE analysis of Scots pine (*Pinus sylvestris*) nSSR variation. Prior to the analysis, six loci (PtTX3116, SPAC11_6, psy117, PtTX3107, psy12 and psy125) displaying null allele frequencies above 10% were removed.


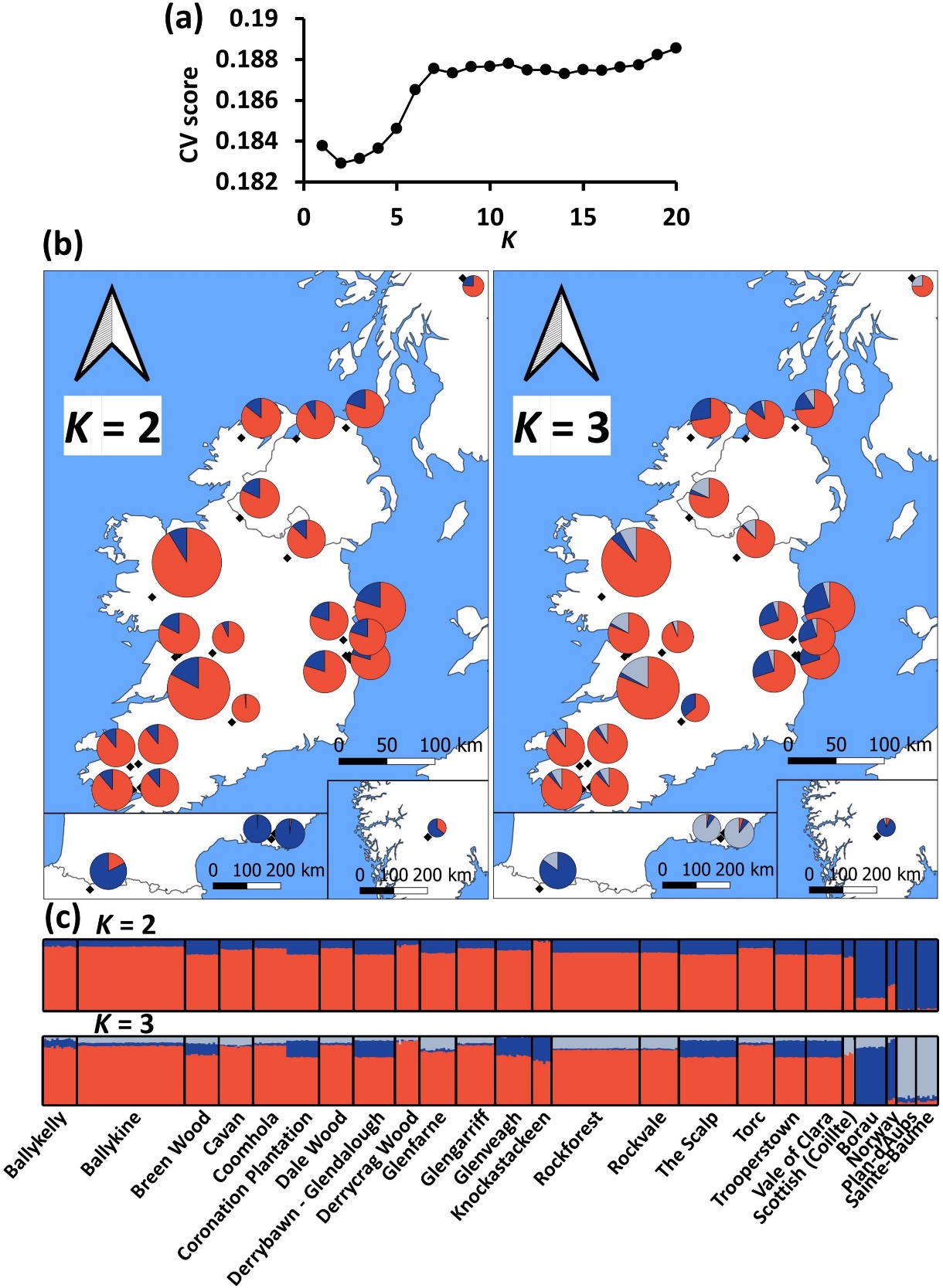


**Figure S13.** Results of an analysis of genotype groups (K) using TESS3. **(a)** Given are the cross-validation (CV) scores based on the root-mean squared errors between the genotypic frequencies predicted from a training set to those computed from a test set (i.e., genotype likelihood) for each locus. **(b)** The frequency of K groups within each population are given, including (inset) populations from Spain, France and Norway (for Norway only three individual are used). **(c)** Individual K ancestral proportions for each individual.


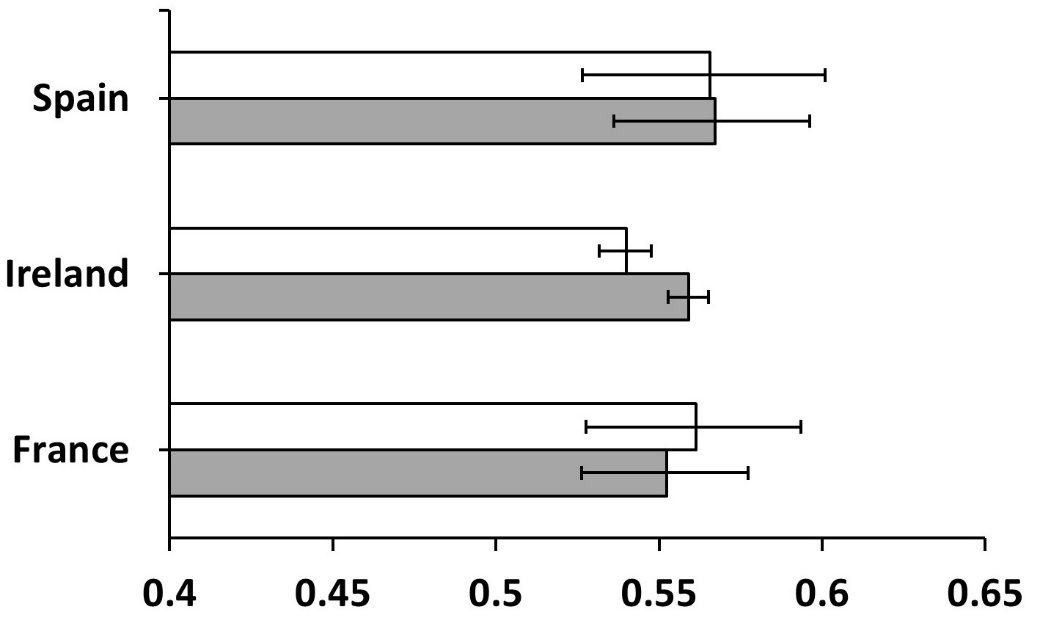


**Figure S14.** Estimates of expected heterozygosity (H_e_) for Pinus sylvestris from three different regions based on 18 nSSR loci (grey) and a reduced set of 12 loci (white). The latter remained after removing six loci (PtTX3116, SPAC11_6, psy117, PtTX3107, psy12 and psy12) which displayed null allele frequencies above 10%. Error bars are 95% CI values derived from 1,000 bootstrap permutations of the data.

**References:**

1. Provan J, Soranzo N, Wilson NJ, McNicol JW, Forrest GI, Cottrell J, et al. Gene-pool variation in caledonian and European Scots pine (*Pinus* *sylvestris* L.) revealed by chloroplast simple-sequence repeats. *Proc* *Biol* *Sci*. 1998;265:1697–705.

2. Wójkiewicz B, Wachowiak W. Substructuring of Scots pine in Europe based on polymorphism at chloroplast microsatellite loci. *Flora - Morphology, Distribution, Functional Ecology of Plants*. 2016;220:142–9.

3. Dering M, Kosiński P, Wyka TP, Pers–Kamczyc E, Boratyński A, Boratyńska K, et al. Tertiary remnants and Holocene colonizers: Genetic structure and phylogeography of Scots pine reveal higher genetic diversity in young boreal than in relict Mediterranean populations and a dual colonization of Fennoscandia. *Diversity* *and* *Distributions*. 2017;23:540–55.

4. Robledo-Arnuncio JJ, Collada C, Alía R, Gil L. Genetic Structure of Montane Isolates of *Pinus* *sylvestris* L. in a Mediterranean Refugial Area. *Journal* *of* *Biogeography*. 2005;32:595–605.

5. Scalfi M, Piotti A, Rossi M, Piovani P. Genetic variability of Italian southern Scots pine (*Pinus* *sylvestris* L.) populations: the rear edge of the range. *Eur* *J* *Forest* *Res*. 2009;128:377.

6. Przybylski P, Tereba A, Meger J, Szyp-Borowska I, Tyburski Ł. Conservation of Genetic Diversity of Scots Pine (*Pinus* *sylvestris* L.) in a Central European National Park Based on cpDNA Studies. *Diversity*. 2022;14:93.

7. Urbaniak L, Wojnicka-Półtorak A, Celiński K, Lesiczka P, Pawlaczyk E, Aučina A. Genetic resources of relict populations of *Pinus* *sylvestris* (L.) in Western Carpathians assessed by chloroplast microsatellites. *Biologia*. 2019;74:1077–86.

8. Semerikov NV, Petrova IV, Sannikov SN, Semerikova SA, Tashev AN, Lascoux M, et al. Cytoplasmic DNA variation does not support a recent contribution of *Pinus* *sylvestris* L. from the Caucasus to the main range. *Tree Genetics & Genomes*. 2020;16:59.

9. Sheller M, Ciocîrlan E, Mikhaylov P, Kulakov S, Kulakova N, Ibe A, et al. Chloroplast DNA Diversity in Populations of *P. sylvestris* L. from Middle Siberia and the Romanian Carpathians. *Forests*. 2021;12:1757.

10. Kavaliauskas D, Danusevičius D, Baliuckas V. New Insight into Genetic Structure and Diversity of Scots Pine (*Pinus* *sylvestris* L.) Populations in Lithuania Based on Nuclear, Chloroplast and Mitochondrial DNA Markers. *Forests*. 2022;13:1179.

11. Semerikov VL, Semerikova SA, Dymshakova OS, Zatsepina KG, Tarakanov VV, Tikhonova IV, et al. Microsatellite loci polymorphism of chloroplast DNA of scots pine (*Pinus* *sylvestris* L.) in Asia and eastern Europe. *Russ J Genet*. 2014;50:577–85.

12. González Díaz P. Development and maintenance of genetic diversity in Scots pine, *Pinus* *sylvestris* (L.). phd. University of Stirling; 2018.

13. Zimmer K, Sønstebø JH. A preliminary study on the genetic structure of Northern European *Pinus* *sylvestris* L. by means of neutral nuclear microsatellite markers. *Scandinavian Journal of Forest Research*. 2018;33:6–13.

14. Belletti P, Ferrazzini D, Piotti A, Monteleone I, Ducci F. Genetic variation and divergence in Scots pine (*Pinus* *sylvestris* L.) within its natural range in Italy. *Eur J Forest Res*. 2012;131:1127–38.

15. Bernhardsson C, Floran V, Ganea SL, García-Gil MR. Present genetic structure is congruent with the common origin of distant Scots pine populations in its Romanian distribution. *Forest Ecology and Management*. 2016;361:131–43.

16. Șofletea N, Mihai G, Ciocîrlan E, Curtu AL. Genetic Diversity and Spatial Genetic Structure in Isolated Scots Pine (*Pinus* *sylvestris* L.) Populations Native to Eastern and Southern Carpathians. *Forests*. 2020;11:1047.

17. Dering M, Baranowska M, Beridze B, Chybicki IJ, Danelia I, Iszkuło G, et al. The evolutionary heritage and ecological uniqueness of Scots pine in the Caucasus ecoregion is at risk of climate changes. *Sci Rep*. 2021;11:22845.

18. Ilinov A, Raevsky B, Chirva O. The state of gene pool of the basic forest-forming species of the white sea watershed (on the example of a *Picea* × *fennica* (Regel) kom. And *Pinus* *sylvestris* L.). *Ecological* *genetics*. 2020;18:185–202.
