# Supplementary material for: Molecular characterisation of Pinus sylvestris (L.) in Ireland at the western limit of the species distribution
Source: BMC Ecol Evol. 2024 Jan 23;24:12. doi: 10.1186/s12862-023-02181-3 (PMC10807061; doi:10.1186/s12862-023-02181-3)
Supplement: Supplementary file 5 — Additional file 5: Figure S12. Individual bar plots showing admixture coefficients for K = 2 to K = 15 ancestral groups derived from a STRUCTURE analysis of Scots pine (Pinus sylvestris) nSSR variation. Prior to the analysis, six loci (PtTX3116, SPAC11_6, psy117, PtTX3107, psy12 and psy125) displaying null allele frequencies above 10% were removed. [file 12862_2023_2181_MOESM5_ESM.pdf]

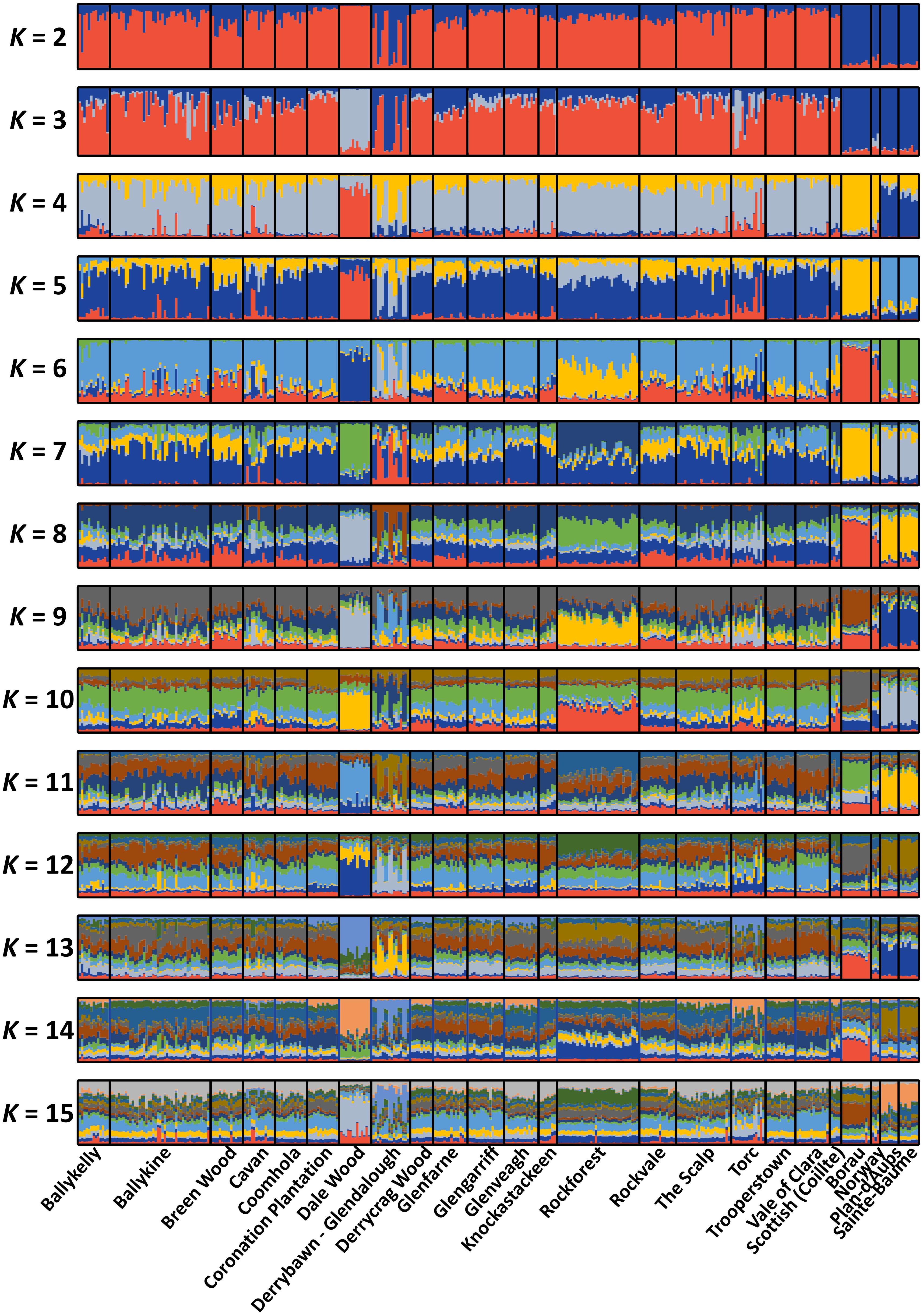

Figure S12. Individual bar plots showing admixture coefficients for K = 2 to K = 15 ancestral groups derived from a STRUCTURE analysis of Scots pine (*Pinus sylvestris*) nSSR variation. Prior to the analysis, six loci (PtTX3116, SPAC11_6, psy117, PtTX3107, psy12 and psy125) displaying null allele frequencies above 10% were removed.
